# Preoperative versus postoperative single-fraction or fractionated stereotactic radiosurgery for resected brain metastases: a systematic review and network meta-analysis

**DOI:** 10.3389/fonc.2026.1848924

**Published:** 2026-10-14

**Authors:** Tian Zhao, Shan Lu

**Affiliations:** Department of Neurosurgery, First Affiliated Hospital of Jilin University, Changchun, China

**Keywords:** brain metastases, leptomeningeal disease, network meta-analysis, preoperative SRS, radiation necrosis, stereotactic radiosurgery

## Abstract

**Background:**

The optimal timing and fractionation of perioperative stereotactic radiotherapy for resected brain metastases remain uncertain. We evaluated the comparative evidence for preoperative and postoperative stereotactic treatment and the validity of indirect comparisons.

**Methods:**

We searched PubMed/MEDLINE, CENTRAL, Web of Science Core Collection, and ClinicalTrials.gov for comparative studies published or posted through 12 August 2026. Primary outcomes were local control or failure, leptomeningeal disease (LMD), and radiation necrosis (RN). Compatibility for quantitative synthesis was assessed by treatment definition, analysis unit, outcome definition, effect measure, and estimation method. Compatible patient-level hazard ratios (HRs) for local failure were combined in an exploratory fixed-effect network model. Incompatible evidence and patient-level and lesion-level estimates were synthesized separately. Risk of bias and certainty of evidence were also assessed.

**Results:**

Sixteen comparative studies were included, comprising six randomized trials and ten non-randomized studies. Three studies provided compatible patient-level HRs and formed a four-treatment local-failure network. Postoperative single-fraction stereotactic radiosurgery (SRS) reduced local failure compared with observation in one randomized trial (HR, 0.46; 95% CI, 0.24–0.88). Estimates for preoperative versus postoperative single-fraction SRS (HR, 1.57; 95% CI, 0.38–6.49) and postoperative single-fraction SRS versus three- to five-fraction treatment (HR, 0.82; 95% CI, 0.09–7.32) were imprecise. All indirect comparisons had wide confidence intervals that included 1. Each direct comparison was informed by one study, and the network contained no closed loop. Some indirect estimates were sensitive to the evidence selected for the single-fraction versus fractionated-treatment comparison. Evidence for LMD and RN did not form compatible connected networks and was synthesized descriptively. Certainty was moderate for postoperative single-fraction SRS versus observation and very low for the remaining local-failure comparisons.

**Conclusions:**

Randomized evidence supports postoperative single-fraction SRS over observation for reducing local failure. However, current evidence does not establish a reliable hierarchy among preoperative SRS, postoperative single-fraction SRS, and postoperative fractionated treatment. Evidence for LMD and RN remains insufficient for network comparison. Treatment decisions should account for individual clinical characteristics until adequately powered prospective comparisons become available.

**Systematic review registration:**

https://www.crd.york.ac.uk/PROSPERO/, identifier CRD420261341468.

## 1 Introduction

Brain metastases (BM) are the most common intracranial tumors in adults and affect many patients with systemic cancer (1–3). Advances in systemic therapy and neuroimaging have increased both their detection and the need for durable intracranial control (1, 2). Resection remains important for large, symptomatic, or accessible lesions because it relieves mass effect, provides tissue for diagnosis, and supports local disease control (1–3). However, surgery alone is associated with a substantial risk of local recurrence (4).

Whole-brain radiotherapy (WBRT) was historically used after resection to improve local and distant intracranial control (1, 2). Randomized trials later showed that this benefit did not consistently improve overall survival and came at the cost of greater neurocognitive deterioration (5). These findings shifted practice toward postoperative stereotactic radiosurgery (SRS) or stereotactic radiotherapy (SRT) in appropriately selected patients (1–3, 6). Postoperative SRS improves local control compared with observation and better preserves cognitive function than WBRT without compromising overall survival (4, 5).

The optimal timing and fractionation of perioperative stereotactic treatment remain uncertain (3, 7–10). Postoperative single-fraction SRS is widely used, but large or irregular cavities may limit safe dose delivery and increase the risk of radiation necrosis (RN) (3, 6, 7). Leptomeningeal disease (LMD) is another important pattern of failure after resection and focal radiotherapy (7–10). These concerns have prompted interest in preoperative SRS and postoperative fractionated stereotactic radiotherapy (FSRT) (7–10). Preoperative treatment may improve target delineation and reduce the dissemination of viable tumor cells during surgery (8–10). Fractionation may allow larger cavities to be treated while reducing the dose delivered per fraction to surrounding brain tissue (3, 6, 7). Whether these potential advantages improve clinical outcomes remains unclear (8–10).

Comparative assessment is limited by marked clinical and methodological variation across studies (8–10). Treatment timing, fractionation, patient selection, lesion characteristics, outcome definitions, analysis units, and statistical methods differ substantially (8–10). Patient-level and lesion-level estimates are not necessarily interchangeable, and Kaplan–Meier estimates should not be treated as equivalent to competing-risk cumulative-incidence estimates (11). Combining clinically distinct fractionation schedules within a single treatment category may introduce further bias. Moreover, randomized and non-randomized studies inform different comparisons, which limits transitivity and weakens indirect inference. A connected evidence network therefore does not, by itself, justify treatment ranking.

We conducted a systematic review of perioperative radiotherapy strategies for resected brain metastases. The strategies included preoperative single-fraction SRS, preoperative FSRT, postoperative single-fraction SRS, postoperative FSRT, WBRT, and observation. Clinical and methodological compatibility was assessed separately for local control or failure, LMD, and RN. Exploratory network synthesis was restricted to compatible relative-effect estimates forming a connected network; otherwise, the evidence was synthesized descriptively. We also evaluated study-design differences, transitivity, robustness to alternative evidence pathways, and certainty of evidence.

## 2 Methods

This systematic review was reported in accordance with the PRISMA 2020 statement and its extension for network meta-analyses (PRISMA-NMA) (12, 13). The review was not prospectively registered.

### 2.1 Search strategy and selection criteria

We searched PubMed/MEDLINE, the Cochrane Central Register of Controlled Trials (CENTRAL), Web of Science Core Collection, and ClinicalTrials.gov. The original search covered records published or registered from 1 January 2000 to 31 December 2024. Targeted update searches covered the period from 1 January 2025 to 12 August 2026. The final search date was 12 August 2026. Search terms were adapted for each source and covered brain metastases, surgical resection, stereotactic radiosurgery or radiotherapy, treatment timing, fractionation, and whole-brain radiotherapy. No language restrictions were applied. We also screened the reference lists of eligible studies and relevant systematic reviews. The complete source-specific search strategies, search periods, filters, and retrieval counts are provided in **Supplementary Table S1**.

Eligible studies included adults (≥18 years) with surgically resected brain metastases and compared at least two treatment strategies of interest. These comprised preoperative single-fraction stereotactic radiosurgery (SRS), preoperative fractionated stereotactic radiotherapy (FSRT), postoperative single-fraction SRS, postoperative FSRT, postoperative whole-brain radiotherapy (WBRT), and observation after resection. Fractionated regimens were classified by the reported number of fractions. Three- to five-fraction regimens were combined only when considered clinically comparable for a specific analysis, whereas seven-fraction FSRT was retained as a separate treatment category. Eligible designs included randomized controlled trials, prospective comparative studies, and retrospective comparative cohorts with at least 10 patients or lesions per treatment group. Studies were required to report local control or failure, leptomeningeal disease (LMD), or radiation necrosis (RN).

We excluded single-arm studies, case reports, reviews, editorials, and case series with fewer than 10 patients or lesions per treatment group. We also excluded dosimetric or treatment-planning studies without clinical outcomes. Studies that included unresected lesions were excluded unless data for resection cavities could be extracted separately. Reports were also excluded when patients or lesions could not be assigned to a predefined treatment category. Conference abstracts were considered for qualitative synthesis but excluded from quantitative synthesis when group-specific sample sizes, outcome estimates, or measures of uncertainty were unavailable.

Eligibility for quantitative synthesis was assessed separately for each outcome. Evidence was combined only when treatment definitions, analysis units, outcome definitions, assessment time points, and effect measures were sufficiently compatible. Patient-level and lesion-level estimates were analyzed separately because multiple lesions within the same patient could not be assumed to be independent. Kaplan–Meier estimates and competing-risk cumulative-incidence estimates were retained as reported and were not converted into estimated event counts (11).

### 2.2 Data extraction and risk-of-bias assessment

Two reviewers independently screened titles and abstracts, assessed full-text reports, and extracted data using a standardized form. Extracted variables included study design, participant and tumor characteristics, treatment details, outcome definitions, analysis unit, follow-up, and effect estimates. Treatment data included radiotherapy timing, dose, fractionation, target definition, and treatment margin. Patient-level and lesion-level data were recorded separately.

We extracted the effect measures reported for each outcome, including hazard ratios (HRs), subdistribution hazard ratios (SHRs), 95% confidence intervals (CIs), and fixed-time Kaplan–Meier or competing-risk estimates. Adjusted estimates were preferred, and the adjustment covariates were recorded. Estimates reported only graphically, or without sufficient information to determine their uncertainty, were excluded from relative-effect synthesis. Fixed-time estimates were retained as reported and were not converted into event counts.

Potential cohort overlap was assessed using participating institutions, recruitment periods, eligibility criteria, and sample characteristics. For each outcome and comparison, we retained the most informative dataset containing the relevant non-duplicated information.

Randomized controlled trials were assessed using the revised Cochrane Risk of Bias 2 (RoB 2) tool (34). Non-randomized comparative studies were assessed using the Risk Of Bias In Non-randomized Studies of Interventions (ROBINS-I) tool (14). Outcome-level assessments were performed when sufficient information was available. Disagreements concerning study selection, data extraction, and risk-of-bias judgments were resolved through discussion and consensus between the two reviewers.

### 2.3 Outcome definitions

The primary outcomes were local control (LC) or local failure, leptomeningeal disease (LMD), and radiation necrosis (RN). Definitions and assessment methods were extracted as reported and evaluated for comparability before quantitative synthesis.

LC was defined as the absence of radiographic progression within or immediately adjacent to the treated resection cavity, according to each study. When local failure was reported, its original direction was retained in relative-effect analyses. Time-to-local-failure effects were expressed as HRs with 95% CIs. For absolute-risk reporting, 12-month LC was calculated as one minus the reported 12-month local-failure risk only when the two outcomes were complementary within the same estimation framework.

LMD was defined by radiographic evidence on magnetic resonance imaging, cytological confirmation in cerebrospinal fluid, or both. Classical or diffuse LMD and nodular meningeal disease were recorded separately and were not considered interchangeable. Kaplan–Meier and competing-risk estimates were retained in their reported form.

RN was classified according to the diagnostic criteria used in each study, including radiographic, clinical, or pathological evidence. Any-grade radiographic RN, symptomatic RN, and grade ≥2 RN were extracted as separate outcome categories. Differences in diagnostic criteria and severity definitions were considered when determining eligibility for quantitative synthesis. Diagnostic methods, including serial or advanced imaging, corticosteroid requirement, surgery, and pathological confirmation, were recorded when available.

The principal time point for absolute-risk reporting was 12 months. Estimates at other time points were retained for descriptive synthesis but were not combined with 12-month estimates. Eligibility for quantitative synthesis also considered the outcome definition, analysis unit, estimation method, and effect measure.

### 2.4 Statistical analysis

#### 2.4.1 Assessment of evidence structure and effect measures

The feasibility of quantitative synthesis was assessed separately for LC or local failure, LMD, and RN. Studies were mapped by treatment category, outcome definition, effect measure, analysis unit, and assessment time point. For each outcome, we examined network connectivity, the number of studies informing each direct comparison, and the presence of closed loops (15). Patient-level and lesion-level estimates were analyzed separately. HRs and SHRs were also treated as distinct effect measures. For local failure, an HR below 1 favored the first-listed treatment.

Pairwise meta-analysis was considered when at least two independent studies reported compatible estimates for the same outcome and treatment comparison. No comparison met this criterion within the prespecified primary evidence sets. Twelve-month Kaplan–Meier and competing-risk estimates were therefore summarized descriptively by outcome, treatment, analysis unit, and estimation method. Within-study absolute risk differences were calculated from the reported 12-month risks but were not pooled across studies.

#### 2.4.2 Exploratory network model

Compatible patient-level HRs for local failure formed a connected network of four treatments: preoperative single-fraction SRS (A), postoperative single-fraction SRS (B), postoperative three- to five-fraction FSRT/SRS (C), and observation (E). The network contained three single-study direct comparisons and no closed loop. We therefore fitted an exploratory fixed-effect, contrast-based consistency model on the log-HR scale (15). Log-HRs were weighted by inverse variance, with standard errors derived from the reported 95% CIs. Estimates were exponentiated and reported as HRs with 95% CIs.

Evidence outside this connected, clinically compatible component was not incorporated by merging distinct fractionation schedules, analysis units, or effect measures. Postoperative seven-fraction FSRT versus WBRT formed a separate evidence component and was reported independently. Compatible relative effects for LMD and RN did not form connected networks and were synthesized descriptively. Treatment rankings, P-scores, and SUCRA values were not calculated because the sparse, tree-shaped network did not support a meaningful treatment hierarchy.

#### 2.4.3 Heterogeneity, consistency, and transitivity

Between-study heterogeneity could not be estimated from the primary local-failure network because each direct comparison included one study. The absence of a closed loop also precluded node-splitting and design-by-treatment interaction analyses. Heterogeneity and disagreement between direct and indirect evidence were therefore classified as not estimable or not applicable. These classifications were not interpreted as evidence of homogeneity or consistency.

Transitivity was assessed qualitatively by comparing potential effect modifiers across direct comparisons. These included study design, participant selection, analysis unit, lesion or cavity volume, radiation dose and fractionation, treatment timing, follow-up, systemic therapy or immunotherapy, primary tumor distribution, outcome definition, and time-to-event method. Differences in these characteristics were considered when interpreting indirect estimates.

#### 2.4.4 Robustness, sensitivity, subgroup, and reporting-bias assessments

Leave-one-study-out analyses examined whether removing each contributing study disconnected the local-failure network or removed a treatment node. Directional stability was assessed using the lower and upper limits of the reported 95% CIs for each direct estimate.

Because heterogeneity could not be estimated from single-study network edges, additional uncertainty was examined using prespecified values of τ = 0.10, 0.20, and 0.40 on the log-HR scale. These scenarios assessed how additional between-study uncertainty affected the confidence intervals. They were not treated as empirical estimates of heterogeneity.

We also replaced the primary patient/cavity-level estimate from Eaton et al. with the lesion-level estimate from Kretzschmar et al. for the postoperative single-fraction versus fractionated-treatment edge. The resulting indirect comparisons were then recalculated. Because this substitution changed the analysis unit, it was considered a stress test rather than an alternative primary model.

In a separate sensitivity analysis, estimates from Eaton et al. and Kretzschmar et al. were aligned to the same treatment direction. They were then combined using conventional fixed-effect and random-effects pairwise models. This analysis deliberately combined patient/cavity-level and lesion-level evidence and did not replace the primary evidence set. Heterogeneity estimates were interpreted cautiously because only two studies were included and their analysis units differed.

Planned RCT-only and clinical subgroup analyses were assessed for feasibility. They were not quantitatively synthesized because the available evidence did not retain a connected network or provide compatible subgroup-specific relative effects. The proposed subgroups included large lesions and radioresistant primary tumors. Funnel plots and regression-based tests were not used because no outcome-specific synthesis included enough independent studies to assess small-study effects reliably (16).

Analyses were conducted in R version 4.5.3. Network geometry was examined using igraph version 2.3.3, and figures were generated using ggplot2 version 4.0.3. Fixed-effect network calculations and robustness analyses were implemented using the reproducible study-level code provided in the Supplementary Materials.

### 2.5 Certainty of evidence

Certainty was evaluated separately for each direct and indirect comparison using an adapted GRADE framework informed by CINeMA domains (17, 18). The assessment considered within-study bias, reporting bias, indirectness, imprecision, heterogeneity, and incoherence. Certainty was classified as high, moderate, low, or very low.

Direct-evidence judgments considered study design, the relevant RoB 2 or ROBINS-I assessment, clinical applicability, and precision. Indirect-evidence judgments were informed by the certainty of the contributing direct evidence. Further downgrading was applied for concerns about transitivity, indirectness, or dependence on a sparse evidence pathway.

Incoherence could not be evaluated statistically because the local-failure network contained no closed loop. Heterogeneity also could not be estimated for comparisons informed by a single study. These domains were recorded as not applicable or not estimable rather than interpreted as showing no concern.

Two reviewers independently assessed certainty at the comparison level, and disagreements were resolved through discussion and consensus. The rationale for each domain judgment and overall rating was recorded. CINeMA software was not used because the sparse network did not support a conventional network-level assessment. The resulting structured comparison-level judgments are presented in **Supplementary Table S9**.

## 3 Results

### 3.1 Study selection and characteristics

The archived screening records documented 415 records identified through the original search. Duplicate removal and title and abstract exclusions were not recorded separately; these processes therefore accounted for a combined total of 356 records. Fifty-nine full-text reports were assessed, of which 43 failed to meet the prespecified eligibility criteria. Sixteen comparative studies were included in the systematic review (**Figure 1**) (4, 5, 19–32).

**Figure 1:**
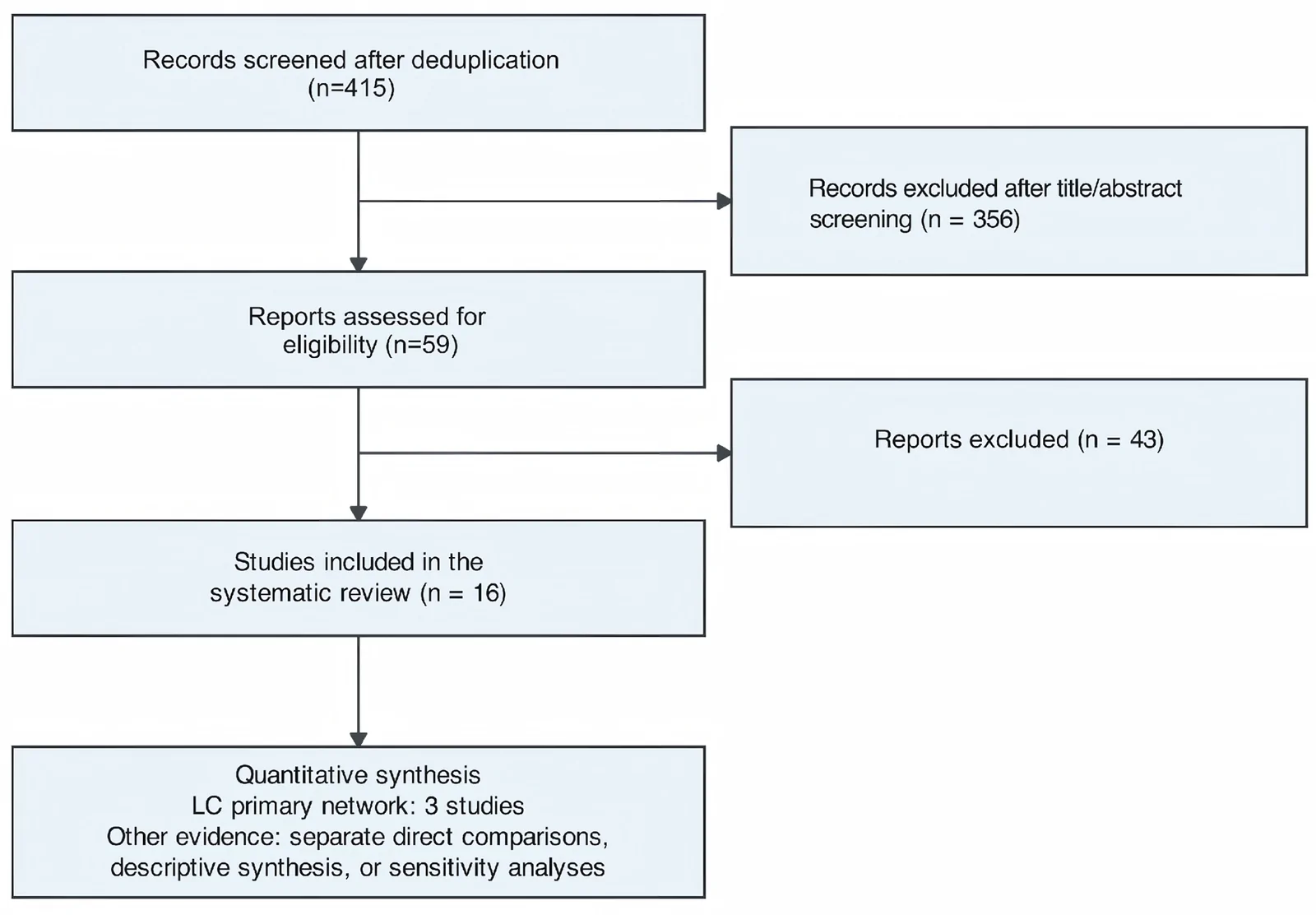
Study-selection flow diagram. Flow diagram showing the identification, screening, eligibility assessment, and inclusion of studies in the systematic review. Database searches, including targeted update searches, identified 415 records. After deduplication and title/abstract screening, 59 full-text reports were assessed for eligibility, of which 43 were excluded, resulting in 16 comparative studies included in the systematic review. Three studies contributed compatible data to the primary local-failure network; the remaining studies contributed to separate direct comparisons, descriptive syntheses, or sensitivity analyses. The archived search records did not separately distinguish duplicate removal from title/abstract exclusions.

The included evidence comprised six randomized controlled trials and ten non-randomized comparative studies, including prospective, propensity-score-matched, and retrospective cohorts. The evaluated strategies were preoperative single-fraction SRS, preoperative fractionated SRT, postoperative single-fraction SRS, postoperative fractionated SRT/SRS, WBRT, and observation. Fractionated regimens ranged from three to seven fractions and were retained as separate categories when they were not considered clinically comparable. Patient-level and lesion-level evidence was analyzed separately.

The 16 studies did not contribute to a single quantitative model. Three studies reported compatible patient-level HRs for local failure and formed the exploratory four-treatment network (4, 19, 20). Other studies informed separate direct comparisons, descriptive syntheses, study-design-stratified assessments, or sensitivity analyses. Their analytical roles depended on the treatment comparison, outcome definition, analysis unit, effect measure, and availability of uncertainty estimates. Study characteristics and analytical roles are summarized in **Table 1**.

**Table 1:** Characteristics and analytical roles of the included studies.

Study	Year	Design	Population/sample	Comparison	Analysis unit	Outcome contribution	Role in synthesis
Kocher et al.	2011	Phase III RCT	Surgical subgroup: 160 patients	Postoperative WBRT vs observation	Patient	LC/LMD and intracranial outcomes	Separate direct evidence
Brömme et al.	2013	Retrospective cohort	42 patients; resection cavities	Postoperative single-fraction SRS vs hypofractionated SRT	Patient/cavity	LC; RN	Descriptive evidence
Patel et al.	2014	Retrospective cohort	132 patients; 141 intracranial metastases	Postoperative SRS vs WBRT	Patient/cavity	LC; intracranial outcomes	Separate direct evidence
Eaton et al.	2015	Retrospective cohort	75 patients; 76 cavities ≥3 cm	Postoperative single-fraction SRS vs 3–5-fraction SRT	Patient/cavity	Local failure; RN	LC primary network
Patel et al.	2015	Multicenter retrospective cohort	180 patients; 189 resected metastases	Preoperative SRS vs postoperative SRS	Patient	LC; LMD; RN	Sensitivity evidence; overlapping cohort assessed
Patel et al.	2016	Multi-institutional retrospective cohort	102 patients; 113 resected metastases	Preoperative SRS vs postoperative SRS/WBRT	Patient	Intracranial outcomes	Descriptive evidence; overlap assessed
Brown et al.	2017	Phase III RCT	194 patients	Postoperative single-fraction SRS vs WBRT	Patient	LC; LMD; RN; survival	Separate direct evidence
Mahajan et al.	2017	Phase III RCT	132 randomized patients	Postoperative single-fraction SRS vs observation	Patient	Local recurrence; LMD	LC primary network
Hong et al.	2019	Phase III RCT	215 patients; mixed local-treatment population	WBRT vs observation	Patient	Intracranial outcomes	Resected-subgroup evidence only
Mallela et al.	2024	Prospective propensity-matched cohort	26 preoperative and 30 postoperative SRS patients	Preoperative vs postoperative single-fraction SRS	Patient	LC; LMD; adverse radiation effect	LC primary network
Kutuk et al.	2024	Retrospective cohort	183 patients; 234 lesions ≥2 cm	Preoperative single-fraction SRS vs postoperative 5-fraction FSRT and other paradigms	Lesion	LF; LMD; RN	Large-lesion sensitivity analysis
Kretzschmar et al.	2025	Retrospective cohort	161 patients; 185 resected metastases	Postoperative single-fraction SRS vs fractionated SRT	Lesion/cavity	LC; RN	Cross-analysis-unit sensitivity
El Shafie et al. (ESTRON)	2025	Phase II RCT	56 randomized; 54 evaluable	Postoperative 7-fraction HFSRT vs WBRT	Patient	LC; LMD; intracranial PFS	Separate network component
Yeboa et al.	2025	Phase III RCT	103 randomized patients	Preoperative vs postoperative SRT (1–5 fractions)	Patient	Perioperative safety and feasibility	Qualitative safety synthesis; oncological outcomes immature
Minniti et al.	2026	Retrospective comparative cohort	190 patients; 202 resected metastases	Postoperative 3-fraction vs 5-fraction SRS	Patient/lesion	LC; symptomatic RN; LMD	Fractionation-specific descriptive analysis
Perlow et al.	2026	Retrospective comparative cohort	458 patients; 534 resected metastases	Preoperative vs postoperative 3–5-fraction FSRT	Lesion	LF; nodular meningeal disease; grade ≥2 RN	Extension and sensitivity analysis

### 3.2 Evidence network and feasibility of quantitative synthesis

Eligibility for quantitative synthesis was assessed separately for LC or local failure, LMD, and RN. Compatibility was determined by treatment timing and fractionation, analysis unit, outcome definition, assessment time point, and effect measure. Patient-level and lesion-level estimates were not combined. Kaplan–Meier and competing-risk estimates were retained in their reported form.

For local failure, three studies reported compatible HRs connecting four treatments: preoperative single-fraction SRS, postoperative single-fraction SRS, postoperative three- to five-fraction FSRT/SRS, and observation (4, 19, 20). The network comprised three single-study direct comparisons and formed a connected tree without a closed loop (**Figure 2**). This structure allowed exploratory indirect estimation. However, between-study heterogeneity could not be estimated, and disagreement between direct and indirect evidence could not be tested.

**Figure 2:**
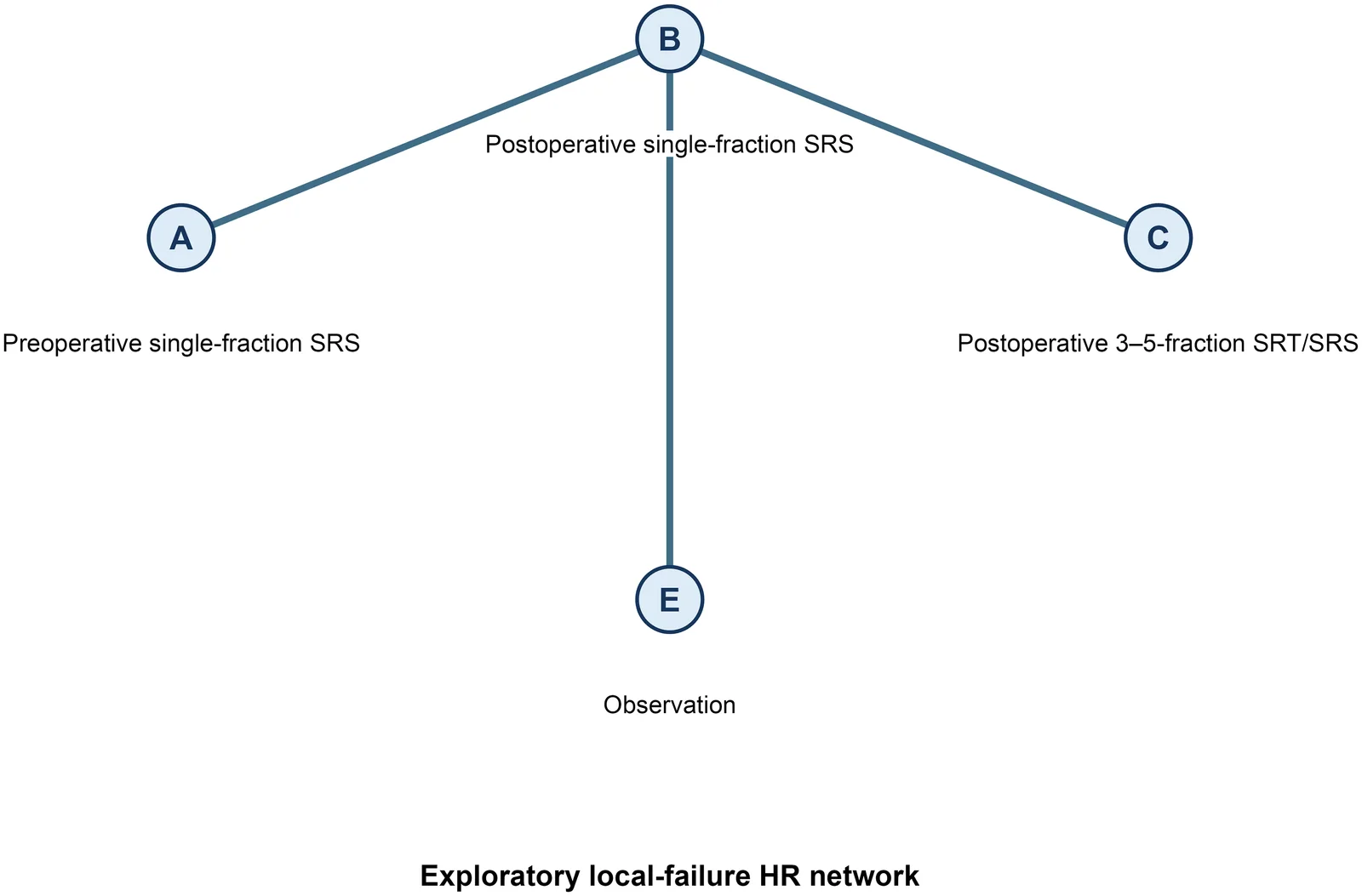
Exploratory evidence network for local failure. The network included four treatment categories: preoperative single-fraction stereotactic radiosurgery (SRS; A), postoperative single-fraction SRS (B), postoperative three- to five-fraction fractionated stereotactic radiotherapy/SRS (SRT/SRS; C), and observation (E). Three studies provided compatible hazard ratios (HRs) for local failure, resulting in three direct comparisons and a connected tree-shaped network without a closed loop. Each edge was informed by a single study. Edge width is uniform and does not represent sample size, precision, treatment efficacy, or treatment ranking. Because the network contained no closed loop, agreement between direct and indirect evidence could not be assessed. SRS, stereotactic radiosurgery; SRT, stereotactic radiotherapy.

Other LC comparisons were excluded from the primary network. Postoperative seven-fraction FSRT versus WBRT formed a separate evidence component and was analyzed independently (21). Comparisons involving preoperative FSRT, postoperative three- versus five-fraction SRS, or lesion-level outcomes could not be connected without merging distinct fractionation categories or analysis units. These data were retained for descriptive or sensitivity analyses.

For LMD, studies differed in their treatment comparisons, outcome definitions, effect measures, and analysis units. Overall LMD and nodular meningeal disease were treated as distinct outcomes, and patient-level estimates were not combined with lesion-level estimates. The compatible relative effects did not form a connected network. Consequently, neither pairwise nor network meta-analysis was performed.

For RN, any-grade radiographic RN, symptomatic RN, and grade ≥2 RN were treated as distinct outcomes. Comparability was further limited by differences in treatment comparisons, effect measures, follow-up, and analysis units. No connected network of compatible relative effects was identified. RN was therefore synthesized descriptively, with selected cross-analysis-unit comparisons examined only in sensitivity analyses.

### 3.3 Local failure

Three studies contributed compatible HRs to the exploratory local-failure network. In the contributing randomized trial, postoperative single-fraction SRS was associated with a lower hazard of local failure than observation (HR, 0.46; 95% CI, 0.24–0.88) (4). The direct estimates for preoperative versus postoperative single-fraction SRS (HR, 1.57; 95% CI, 0.38–6.49) (19) and postoperative single-fraction SRS versus postoperative three- to five-fraction FSRT/SRS (HR, 0.82; 95% CI, 0.09–7.32) (20) were imprecise, with confidence intervals spanning effects in either direction.

The exploratory network provided three indirect estimates. The HR was 1.29 (95% CI, 0.09–17.49) for preoperative single-fraction SRS versus postoperative three- to five-fraction FSRT/SRS, 0.72 (95% CI, 0.15–3.44) for preoperative single-fraction SRS versus observation, and 0.56 (95% CI, 0.06–5.51) for postoperative three- to five-fraction FSRT/SRS versus observation. All confidence intervals were wide and included 1, providing no clear evidence of differences between treatments.

Direct and indirect estimates are presented in **Figure 3** and **Table 2**. Treatment rankings, P-scores, and SUCRA values were not calculated because each direct comparison was informed by one study, the network contained no closed loop, and most estimates were highly imprecise.

**Figure 3:**
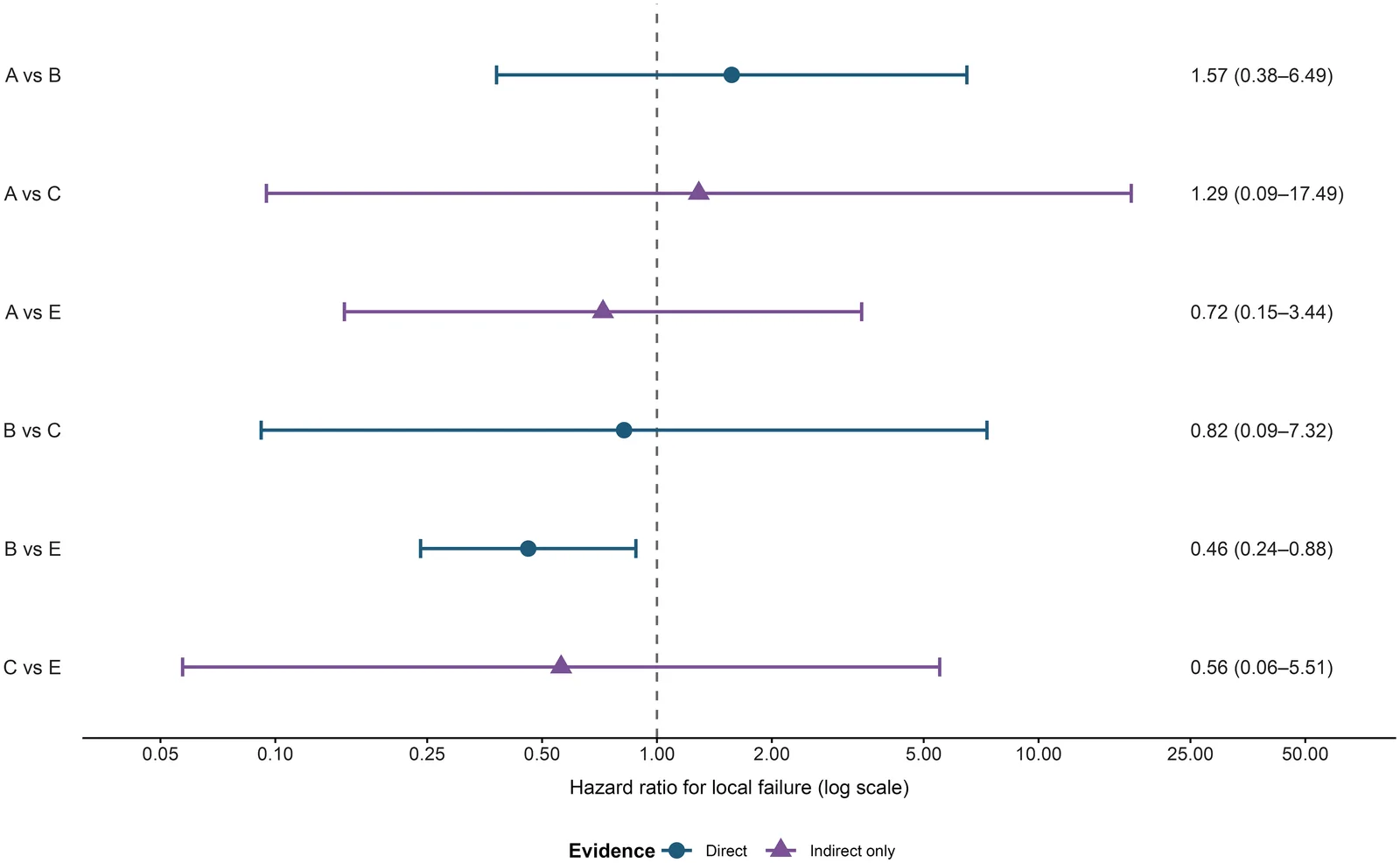
Direct and indirect relative-effect estimates for local failure in the exploratory network. Forest plot showing hazard ratios (HRs) and 95% confidence intervals (CIs) for the three direct and three indirectly estimated treatment comparisons in the exploratory local-failure network. Direct comparisons were each informed by a single study, whereas indirect estimates were derived entirely through the corresponding paths in the tree-shaped network. HR <1 favors the first-listed treatment. No pooled heterogeneity or inconsistency estimate was available because each direct comparison was informed by a single study and the network contained no closed loop. Treatment rankings, P-scores, and SUCRA values were not calculated because of the sparse network structure and substantial imprecision of most estimates. A, preoperative single-fraction SRS; B, postoperative single-fraction SRS; C, postoperative three- to five-fraction SRT/SRS; E, observation; CI, confidence interval; HR, hazard ratio; SRS, stereotactic radiosurgery; SRT, stereotactic radiotherapy.

**Table 2:** Matrix of direct and exploratory indirect hazard-ratio estimates for local failure.

Row treatment	A	B	C	E
A	**—**	1.57 (0.38–6.49)	1.29 (0.09–17.49)	0.72 (0.15–3.44)
B	0.64 (0.15–2.63)	**—**	0.82 (0.09–7.32)	0.46 (0.24–0.88)
C	0.78 (0.06–10.55)	1.22 (0.14–10.89)	**—**	0.56 (0.06–5.51)
E	1.38 (0.29–6.59)	2.17 (1.14–4.16)	1.78 (0.18–17.50)	**—**

A, Preoperative single-fraction SRS, B, Postoperative single-fraction SRS; C, Postoperative three- to five-fraction FSRT/SRS; E, Observation after resection. HR < 1 favors the row treatment; HR > 1 favors the column treatment; Blue cells represent direct evidence, each informed by one study; grey cells represent indirect-only estimates. The network contained four treatments, three direct comparisons and no closed loop. Heterogeneity and inconsistency were not estimable; Treatment ranking was not performed. Reciprocal cells are algebraic inverses of the same model contrast and do not represent independent estimates.

### 3.4 Twelve-month absolute risks

Twelve-month risks were summarized separately by outcome, analysis unit, and estimation method (**Supplementary Figure S1**; **Supplementary Tables S2**, **S3**). Kaplan–Meier and competing-risk estimates were retained as reported and were neither pooled nor converted into event counts.

For local failure, patient-level competing-risk estimates ranged from 2.0% to 27.2%, and patient-level Kaplan–Meier estimates ranged from 17.5% to 57.0%. Lesion-level competing-risk estimates ranged from 2.4% to 9.8%. In the Mahajan randomized trial, the risks were 57% with observation and 28% with postoperative single-fraction SRS, an absolute between-group difference of 29 percentage points. Mallela et al (4). reported Kaplan–Meier estimates of 22.8% after preoperative and 17.5% after postoperative single-fraction SRS, a difference of 5.3 percentage points (19). Competing-risk estimates were 25.6% versus 27.2% in Eaton et al. (20), 2.0% versus 5.3% in the ESTRON trial (21), and 7.0% versus 8.0% in the lesion-level analysis by Kutuk et al. (22). The corresponding absolute differences were 1.6, 3.3, and 1.0 percentage points, respectively.

For LMD, patient-level 12-month cumulative-incidence estimates ranged from 3.2% to 28.0%, and lesion-level estimates ranged from 9.0% to 12.0%. The Mahajan trial reported risks of 16% with observation and 28% with postoperative single-fraction SRS, a difference of 12 percentage points (4). In the ESTRON trial, the risks were 27.0% after postoperative seven-fraction FSRT and 8.7% after WBRT, a difference of 18.3 percentage points (21). Patel et al. reported risks of 3.2% after predominantly single-fraction preoperative SRS and 8.3% after mixed postoperative SRS regimens, a difference of 5.1 percentage points (23). The corresponding differences were 0.3 percentage points between postoperative three- and five-fraction SRS in Minniti et al. (24)and 3.0 percentage points in the lesion-level comparison by Kutuk et al. (22). Nodular meningeal disease was treated separately and was not included in these ranges.

For RN, patient-level 12-month estimates ranged from 1.5% to 19.2%, and lesion-level estimates ranged from 3.0% to 7.8%. Eaton et al. reported any-grade RN risks of 10.3% after postoperative three- to five-fraction FSRT/SRS and 19.2% after postoperative single-fraction SRS, a difference of 8.9 percentage points (20). The corresponding symptomatic RN risks were 6.8% and 16.4%, a difference of 9.6 percentage points. Patel et al. reported symptomatic RN risks of 1.5% after predominantly single-fraction preoperative SRS and 14.6% after mixed postoperative SRS, a difference of 13.1 percentage points (23). The absolute differences were 3.0 percentage points between postoperative three- and five-fraction SRS in Minniti et al. (24), 2.0 percentage points in Kutuk et al. (22), and 1.0 percentage point for grade ≥2 RN in Perlow et al (25).

These risks and within-study differences were treated as study-specific descriptive estimates, not pooled comparative effects. Cross-study comparisons were avoided because study design, treatment definition, analysis unit, outcome definition, and time-to-event method differed.

### 3.5 Study-design-stratified evidence

Relative-effect estimates were examined by study design and analysis unit (**Figure 4**; **Supplementary Table S4**). Randomized evidence was available for postoperative single-fraction SRS versus observation (4), postoperative single-fraction SRS versus WBRT (5), and postoperative seven-fraction FSRT versus WBRT (21). A further randomized comparison of preoperative and postoperative SRT primarily contributed feasibility and safety data because mature comparative oncological outcomes were unavailable (26). Differences in treatments, outcomes, and effect measures prevented these trials from forming a common evidence network. The only randomized comparison included in the exploratory local-failure network favored postoperative single-fraction SRS over observation (HR, 0.46; 95% CI, 0.24–0.88) (4).

**Figure 4:**
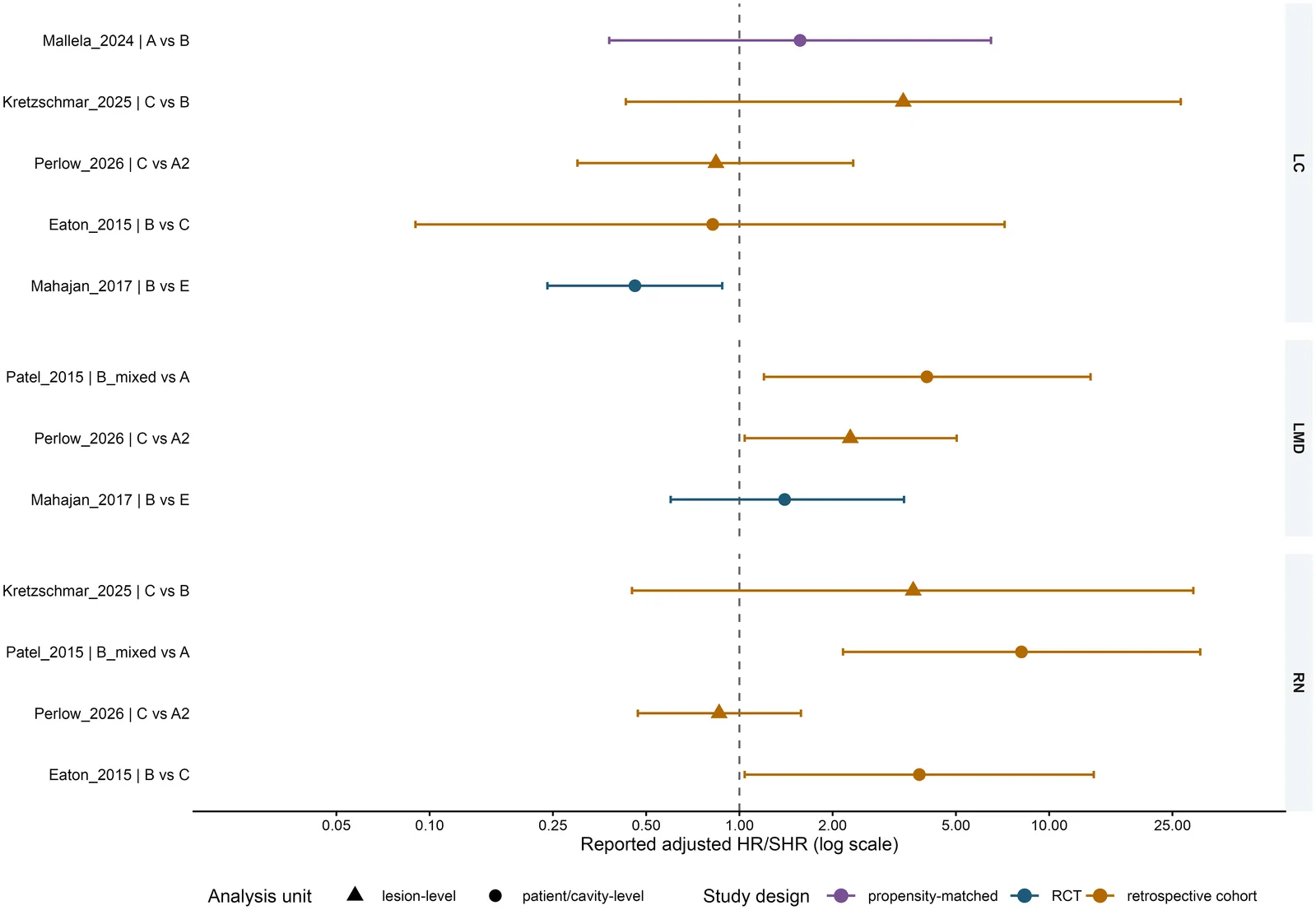
Study-design-stratified relative-effect evidence for local failure, leptomeningeal disease, and radiation necrosis. Study-level relative-effect estimates and 95% confidence intervals are presented according to study design and analysis unit for local failure (LF), leptomeningeal disease (LMD), and radiation necrosis (RN). Randomized controlled trials, propensity-score-matched studies, and retrospective comparative cohorts are displayed separately to illustrate differences in the design and evidential basis of the available comparisons. Patient-level, patient/cavity-level, and lesion-level estimates were retained separately and were not pooled across analysis units. Effect estimates are presented in the treatment direction reported for each comparison; therefore, the direction and definition of each comparison should be considered when interpreting the HR or subdistribution HR (SHR). The image provides a structured display of study-specific comparative evidence rather than pooled treatment effects. CI, confidence interval; FSRT, fractionated stereotactic radiotherapy; HR, hazard ratio; LF, local failure; LMD, leptomeningeal disease; RN, radiation necrosis; SHR, subdistribution hazard ratio; SRS, stereotactic radiosurgery; SRT, stereotactic radiotherapy; WBRT, whole-brain radiotherapy. Study-level estimates are stratified by design and analysis unit and are not pooled across outcomes, estimands or nodes. The display assesses directional concordance; it is not a pooled meta-analysis.

The propensity-score-matched comparison of preoperative and postoperative single-fraction SRS showed no clear difference in local failure (HR, 1.57; 95% CI, 0.38–6.49) (19). Matching reduced measured baseline imbalance, but differences in treatment volume and residual treatment-selection confounding remained possible. This evidence was therefore less certain than the randomized comparison of postoperative SRS with observation.

Comparisons of postoperative single-fraction and fractionated SRT/SRS were derived from retrospective cohorts. Eaton et al. reported an imprecise patient/cavity-level estimate (HR, 0.82; 95% CI, 0.09–7.32) (20). The planning-target-volume-adjusted lesion-level estimate from Kretzschmar et al. was also imprecise (HR for fractionated versus single-fraction SRT/SRS, 3.38; 95% CI, 0.43–26.60) (27). Differences in analysis unit, fractionation selection, and cavity volume limited comparison of these estimates.

Across study designs, the evidence did not show a consistent advantage of preoperative over postoperative stereotactic treatment or of single-fraction over fractionated postoperative treatment. Randomized evidence supported postoperative single-fraction SRS over observation for local failure. Other comparisons relied mainly on non-randomized evidence with wide confidence intervals. Patient-level and lesion-level estimates were displayed separately and were not treated as mutually confirmatory. Study-design-stratified LMD and RN findings are also shown in **Figure 4** and **Supplementary Table S4** but were not pooled.

### 3.6 Robustness, transitivity, and evidence-path analyses

Each direct comparison in the local-failure network was informed by one study. Postoperative single-fraction SRS versus observation was informed by a randomized trial, preoperative versus postoperative single-fraction SRS by a propensity-score-matched study, and postoperative single-fraction SRS versus fractionated treatment by a retrospective cohort. The three indirect comparisons therefore depended entirely on pathways through this tree-shaped network.

The complete network was structurally dependent on all three studies. Removing Mahajan et al. disconnected observation, removing Mallela et al. disconnected preoperative single-fraction SRS, and removing Eaton et al. disconnected postoperative fractionated treatment. Removal of any study prevented re-estimation of the complete four-treatment network and its indirect comparisons.

Only postoperative single-fraction SRS versus observation retained the same effect direction across its reported 95% CI. The intervals for the other two direct comparisons and all three indirect comparisons crossed 1. Their estimated directions were therefore not stable across the uncertainty represented by the contributing effects.

Prespecified heterogeneity scenarios progressively widened the confidence intervals as τ increased from 0 to 0.10, 0.20, and 0.40 on the log-HR scale, without changing the point estimates. For postoperative single-fraction SRS versus observation, the interval remained below 1 at τ = 0.10 and 0.20 but crossed 1 at τ = 0.40 (HR, 0.46; 95% CI, 0.17–1.27). All other direct and indirect estimates included 1 under every scenario. These scenarios assessed robustness and were not empirical estimates of heterogeneity.

Replacing the patient/cavity-level estimate from Eaton et al. with the lesion-level estimate from Kretzschmar et al. preserved network connectivity but changed two derived effect directions. The HR for preoperative single-fraction SRS versus postoperative fractionated SRT/SRS changed from 1.29 to 0.46. The HR for postoperative fractionated SRT/SRS versus observation changed from 0.56 to 1.55. The indirect estimates were therefore sensitive to the study and analysis unit used for this evidence edge.

A separate cross-analysis-unit stress test aligned and combined the Eaton and Kretzschmar estimates. The fixed-effect estimate for postoperative single-fraction SRS versus postoperative fractionated SRT/SRS was 0.48 (95% CI, 0.11–2.14). The conventional random-effects estimate was unchanged (Q = 0.44, P = 0.507; τ² = 0; I² = 0%). These heterogeneity statistics were weakly informative because the analysis included only two studies with different analysis units. This stress test did not replace the primary evidence set.

Potential effect modifiers differed across network edges. These included study design, participant selection, lesion or cavity volume, radiation dose and fractionation, systemic therapy including immunotherapy, follow-up, outcome definition, and time-to-event method. The three edges were informed by a propensity-score-matched cohort, a retrospective cohort of large cavities, and a randomized trial. These differences weakened support for transitivity and reduced confidence in the indirect estimates.

Formal subgroup meta-analysis was not feasible because no prespecified subgroup contained at least two independent studies with a compatible replicated comparison. Findings stratified by study design, analysis unit, treatment timing, fractionation, large-lesion populations, and symptomatic RN were therefore retained as descriptive or sensitivity evidence.

Evidence pathways, leave-one-study-out results, directional-stability assessments, heterogeneity scenarios, sensitivity analyses, potential effect modifiers, and subgroup feasibility are presented in **Supplementary Figure S2** and **Supplementary Tables S5**, **S6**.

### 3.7 Secondary outcomes

Evidence for overall survival, distant intracranial control, and perioperative complications was insufficient for pairwise or network meta-analysis. Studies differed in treatment comparisons, outcome definitions, assessment times, and reported effect measures, leaving too few compatible estimates for pooling.

No comparison showed clear evidence of an overall-survival advantage for one perioperative radiotherapy strategy over another. In the Mahajan randomized trial, postoperative single-fraction SRS did not improve overall survival compared with observation (HR, 1.29; 95% CI, 0.84–1.98). Other randomized comparisons involving focal radiotherapy, WBRT, or observation also did not establish clear survival differences. These studies were not pooled because their treatment regimens, populations, and comparator structures differed.

Distant intracranial control was reported as distant brain failure, intracranial progression, or time to intracranial recurrence. Some randomized comparisons indicated better intracranial disease control with WBRT than with focal treatment or observation, without a demonstrated overall-survival advantage. Postoperative seven-fraction FSRT versus WBRT was retained as a distinct comparison and was not combined with single-fraction or three- to five-fraction postoperative treatment.

The randomized comparison of preoperative and postoperative stereotactic radiotherapy primarily reported perioperative feasibility and safety. Mature comparative LC, LMD, and RN results were unavailable. The limited perioperative and wound-related data showed no clear between-group difference, and definitions varied across studies. No pooled effect was therefore calculated. The trial also reported a shorter interval from randomization to completion of radiotherapy and surgery with preoperative treatment (median, 10 versus 32.5 days; P < 0.001). This was considered a treatment-logistics outcome rather than evidence of oncological efficacy.

Study-level findings for survival, distant intracranial control, perioperative complications, and treatment logistics are summarized in **Supplementary Table S7**.

### 3.8 Risk of bias, small-study effects, and certainty of evidence

All six randomized trials were judged to have some concerns under RoB 2 (**Supplementary Figure S3A**; **Supplementary Table S8**). Concerns primarily involved missing outcome data and outcome measurement. Individual trials also raised concerns about randomization or selection of reported results. All ten non-randomized comparative studies were judged to have a serious overall risk of bias under ROBINS-I (**Supplementary Figure S3B**; **Supplementary Table S8**). The principal concern was residual confounding related to treatment selection, with additional concerns about outcome measurement, missing data, or selective reporting in some studies.

Small-study effects were not formally assessed. Each direct comparison contained too few studies for informative funnel plots or regression-based tests, and the local-failure network comprised three single-study edges. Between-study heterogeneity could not be estimated empirically within the primary network. The absence of a closed loop also precluded statistical assessment of disagreement between direct and indirect evidence.

Using an adapted GRADE framework informed by CINeMA domains, certainty was rated as moderate for postoperative single-fraction SRS versus observation for local failure. The contributing randomized trial had some concerns under RoB 2, but these were not considered sufficient for downgrading within-study bias. The evidence was downgraded for imprecision. Certainty for all other direct and indirect local-failure comparisons was very low, primarily because of serious within-study bias, indirectness, and substantial imprecision (**Supplementary Table S9**). These structured comparison-level judgments were informed by GRADE and CINeMA domains but were not generated using CINeMA software.

## 4 Discussion

This review found that comparative evidence for perioperative radiotherapy after resection of brain metastases remains sparse and clinically heterogeneous. The clearest finding came from a randomized trial in which postoperative single-fraction SRS reduced local failure compared with observation (4). By contrast, comparisons of preoperative versus postoperative single-fraction SRS (19) and postoperative single-fraction versus fractionated treatment (20) were imprecise. All other network estimates relied entirely on indirect pathways and had wide confidence intervals. Compatible relative effects for LMD and RN did not form connected networks and were synthesized descriptively. Thus, randomized evidence supports postoperative single-fraction SRS over observation for reducing local failure but does not establish a hierarchy among preoperative, postoperative single-fraction, and postoperative fractionated stereotactic approaches.

### 4.1 Interpretation of the local-failure evidence

The most reliable local-failure evidence came from the randomized comparison of postoperative single-fraction SRS with observation. Postoperative SRS reduced the hazard of local failure (HR, 0.46; 95% CI, 0.24–0.88) (4). This result supports postoperative single-fraction SRS over observation but should not be extrapolated to comparisons of treatment timing or fractionation.

The direct comparison of preoperative and postoperative single-fraction SRS came from one propensity-score-matched study and was imprecise (HR, 1.57; 95% CI, 0.38–6.49) (19). Although matching reduced measured baseline differences, variation in treatment volume and residual treatment-selection confounding remained possible. The comparison of postoperative single-fraction SRS with three- to five-fraction FSRT/SRS was similarly based on one retrospective cohort and was highly imprecise (HR, 0.82; 95% CI, 0.09–7.32) (20). Cavity size and other selection factors influenced fractionation, limiting interpretation of this estimate (20, 27).

Comparisons involving preoperative single-fraction SRS, postoperative fractionated treatment, and observation relied entirely on indirect pathways. The network contained no closed loop, so disagreement between direct and indirect evidence could not be assessed. Replacing the patient/cavity-level estimate for single-fraction versus fractionated treatment (20) with a lesion-level estimate (27) changed the direction of two derived effects. This indicated that the indirect results depended on the evidence selected for that network edge. They therefore do not establish superior local control with preoperative SRS or postoperative fractionated treatment.

Preoperative treatment may simplify target delineation around an intact lesion and reduce delays associated with postoperative recovery, referral, or changes in cavity geometry (8, 19, 26). Fractionated treatment may accommodate larger targets while reducing the dose delivered per fraction to surrounding normal brain (3, 6, 7, 24, 25). These potential advantages justify prospective evaluation but do not demonstrate superior efficacy. Direct comparative studies require standardized local-failure definitions, appropriate time-to-event methods, and sufficiently mature follow-up.

### 4.2 Leptomeningeal disease and radiation necrosis

The evidence did not support a treatment hierarchy for LMD or RN. LMD studies differed in treatment timing, fractionation, disease definition, analysis unit, and effect measure, and their relative effects did not form a connected network. Twelve-month estimates also varied substantially. LMD incidence was numerically higher after postoperative single-fraction SRS than after observation in the Mahajan (4) trial and after postoperative seven-fraction FSRT than after WBRT in the ESTRON trial (21). These trials addressed different comparisons and do not show that postoperative stereotactic treatment consistently increases LMD risk.

Observational studies have suggested that preoperative irradiation may reduce LMD by treating viable tumor cells before surgical manipulation and dissemination into the cerebrospinal fluid (8, 10, 23, 33). Some comparisons in this review were compatible with lower LMD risk after preoperative treatment. Interpretation was limited by non-randomized treatment allocation, heterogeneous postoperative regimens, possible cohort overlap, and differing definitions of meningeal dissemination. Moreover, the randomized comparison of preoperative and postoperative SRT had not reported mature comparative LC, LMD, or RN outcomes (26). Reduced intraoperative dissemination therefore remains a plausible mechanism rather than an established clinical benefit.

Interpretation of RN was similarly limited by heterogeneous definitions. Any-grade radiographic RN, symptomatic RN, and grade ≥2 RN were not considered interchangeable. Some retrospective comparisons reported numerically lower 12-month RN risks with fractionated than with single-fraction postoperative treatment (20, 27). However, fractionation was often selected for larger cavities, and the relative-effect estimates were imprecise. Comparisons of postoperative three- versus five-fraction treatment and preoperative versus postoperative FSRT (25) showed small or inconsistent absolute differences. Current evidence therefore does not establish that any treatment timing or fractionation strategy consistently minimizes RN.

Fractionation may facilitate treatment of large or irregular cavities while reducing the dose delivered per fraction to surrounding normal brain (3, 6, 7, 24, 25). RN risk may also vary with target volume, dose, fractionation, previous irradiation, systemic therapy, follow-up, and the method used to distinguish RN from progression (7, 20, 25, 27). These factors differed across studies and may have influenced treatment selection and outcome ascertainment. Future studies should prespecify LMD subtypes and RN severity, account for death as a competing event when appropriate, and report adjusted time-to-event estimates alongside absolute risks.

### 4.3 Clinical implications of treatment timing and fractionation

Treatment should be individualized rather than selected from a statistical ranking. Randomized evidence supports postoperative single-fraction SRS over observation for reducing local failure (4), but does not establish superiority over preoperative or fractionated stereotactic treatment. Treatment timing and fractionation should consider lesion and cavity characteristics, treatment logistics, pathological confirmation, surgical timing, previous irradiation, and the feasibility of postoperative target delineation.

Preoperative SRS may offer clearer target definition and avoid delays associated with postoperative recovery or referral (8, 19, 26). It requires close coordination between neurosurgical and radiation oncology teams and is most applicable when resection is planned and the radiographic diagnosis is sufficiently secure. Urgent surgery, substantial mass effect, clinical deterioration, or diagnostic uncertainty may limit its feasibility. Current evidence does not show that these practical advantages improve local control or reduce LMD.

Postoperative fractionated treatment may be considered for large or irregular cavities when single-fraction treatment would expose substantial normal brain tissue. Fractionation reduces the dose delivered per fraction, but the optimal schedule remains uncertain. Three-, five-, and seven-fraction regimens should not be assumed to be interchangeable (7, 21, 24, 25). In particular, postoperative seven-fraction FSRT versus WBRT formed a separate evidence component (21). Observational comparisons are also susceptible to confounding by indication because larger cavities were more likely to receive fractionated treatment (20, 27).

WBRT may improve intracranial control outside the treated cavity but can adversely affect neurocognitive function and quality of life (2, 5, 28, 29). Its role in selected patients was not the focus of this review, and no indication for WBRT can be inferred from the present analysis.

Overall, randomized evidence supports postoperative single-fraction SRS over observation for reducing local failure. The choice among preoperative treatment, postoperative single-fraction SRS, and postoperative fractionated SRT/SRS remains dependent on clinical context and patient preferences. Shared decision-making should acknowledge the limited comparative evidence and uncertainty regarding relative benefits and harms.

### 4.4 Study-design differences, transitivity, and certainty of evidence

Interpretation of the network estimates requires studies across treatment comparisons to be sufficiently similar in potential effect modifiers. Support for this transitivity assumption was limited. The three network edges were informed by a randomized trial, a propensity-score-matched cohort, and a retrospective cohort. These studies differed in participant selection, lesion or cavity volume, radiation dose and fractionation, systemic therapy, follow-up, outcome definition, and estimation method.

Use of systemic therapy varied across studies and was often incompletely reported. Earlier cohorts largely predated the routine use of immune checkpoint inhibitors and contemporary targeted therapies, including tyrosine kinase inhibitors (TKIs), whereas later cohorts reflect a substantially different therapeutic landscape. These differences may have influenced intracranial control and survival, the risks of LMD and RN, and the choice of preoperative or postoperative treatment. Study-level data were insufficient for adjustment, subgroup analysis, or meta-regression; confounding by systemic therapy and treatment era therefore remains possible.

The randomized comparison of postoperative single-fraction SRS with observation provided the strongest direct evidence in the network but did not address treatment timing or fractionation. Propensity-score matching improved balance in the preoperative versus postoperative comparison, although residual confounding remained possible because treatment volumes were not fully balanced. In the retrospective comparison of postoperative single-fraction and fractionated treatment, cavity size and other clinical factors influenced treatment selection, creating a risk of confounding by indication.

These differences were particularly important in the tree-shaped network. Each edge was informed by one study, and no closed loop allowed assessment of disagreement between direct and indirect evidence. Each indirect comparison therefore depended entirely on its contributing pathway and inherited its limitations. Replacing the patient/cavity-level estimate for one edge with lesion-level evidence changed the direction of two derived effects. Mathematical connectivity alone should not be interpreted as evidence that transitivity was adequately supported.

Certainty was moderate for postoperative single-fraction SRS versus observation for local failure. The contributing trial had some concerns under RoB 2, but these did not warrant downgrading for within-study bias; the evidence was downgraded for imprecision. Certainty was very low for all other direct and indirect local-failure comparisons because of within-study bias, indirectness, and substantial imprecision. Confidence intervals containing the null should not be interpreted as evidence of equivalence, and the evidence does not support a reliable treatment hierarchy.

### 4.5 Strengths and limitations

This review evaluated randomized and non-randomized evidence across the principal perioperative radiotherapy strategies used after resection of brain metastases. Clinically distinct regimens, including preoperative fractionated treatment and postoperative seven-fraction FSRT, were not merged into broader treatment nodes. Eligibility for quantitative synthesis was assessed separately for LC or local failure, LMD, and RN. Patient-level and lesion-level evidence was separated, and Kaplan–Meier and competing-risk estimates were retained in their reported forms. Robustness analyses examined study design, evidence pathways, network dependence, prespecified heterogeneity scenarios, alternative analysis units, and certainty of evidence.

Several limitations should be considered. First, the review was not prospectively registered. Treatment-node definitions and quantitative eligibility criteria were refined during the current reanalysis to address clinical and statistical incompatibilities. These changes are documented in the **Supplementary Methods**, but the absence of prospective registration leaves scope for *post hoc* analytical decisions.

Second, network synthesis was feasible only for local failure. The network contained four treatments connected by three single-study edges and had no closed loop. Heterogeneity and disagreement between direct and indirect evidence could not be estimated. Removing any contributing study disrupted the complete network or removed a treatment node. Replacing one patient/cavity-level estimate with lesion-level evidence changed two indirect effect directions.

Third, two of the three direct comparisons came from non-randomized studies. Differences in treatment selection, lesion or cavity volume, radiation dose and fractionation, systemic therapy, follow-up, and outcome assessment weakened support for transitivity and introduced residual confounding. Potentially overlapping cohorts also restricted which evidence could be combined without double counting patients or lesions.

Fourth, clinical and methodological heterogeneity precluded quantitative synthesis of LMD and RN. Overall and nodular meningeal disease were not considered equivalent. RN definitions ranged from any-grade radiographic changes to symptomatic or grade ≥2 events. Differences in analysis unit and time-to-event method further limited comparability. The evidence was also insufficient for formal subgroup meta-analysis or meaningful assessment of small-study effects.

Finally, most direct and indirect local-failure estimates were highly imprecise and supported by very-low-certainty evidence. The review therefore defines the limits of the current comparative evidence rather than providing a definitive ranking of perioperative radiotherapy strategies.

### 4.6 Conclusions

One randomized trial showed a lower hazard of local failure with postoperative single-fraction SRS than with observation. The available evidence did not establish a reliable hierarchy among preoperative SRS, postoperative single-fraction SRS, and postoperative fractionated SRT/SRS. The remaining direct and indirect local-failure comparisons were imprecise and generally supported by very-low-certainty evidence. Differences in study design and potential effect modifiers further reduced confidence in the indirect estimates.

Clinical and methodological heterogeneity precluded network meta-analysis for LMD and RN, and the available evidence did not identify a consistently preferable strategy for either outcome. Treatment timing and fractionation should be individualized according to lesion and cavity characteristics, surgical timing, diagnostic considerations, previous treatment, expected toxicity, and patient preferences. Adequately powered prospective studies with standardized outcome definitions and appropriate time-to-event methods are needed to compare treatment timing and fractionation directly.

## Data Availability

The original contributions presented in the study are included in the article/**Supplementary Material**. Further inquiries can be directed to the corresponding author.

## References

[1] Le Rhun E Guckenberger M Smits M Dummer R Bachelot T Sahm F . EANO-ESMO clinical practice guidelines for diagnosis, treatment and follow-up of patients with brain metastasis from solid tumours. Ann Oncol. (2021) 32:1332–47. doi: 10.1016/j.annonc.2021.07.016 34364998

[2] Vogelbaum MA Brown PD Messersmith H Brastianos PK Burri S Cahill D . Treatment for brain metastases: ASCO-SNO-ASTRO guideline. J Clin Oncol. (2022) 40:492–516. doi: 10.1200/JCO.21.02314 34932393

[3] Gondi V Bauman G Bradfield L Burri SH Cabrera AR Cunningham DA . Radiation therapy for brain metastases: An ASTRO clinical practice guideline. Pract Radiat Oncol. (2022) 12:265–82. doi: 10.1016/j.prro.2022.02.003 35534352

[4] Mahajan A Ahmed S McAleer MF Weinberg JS Li J Brown P . Post-operative stereotactic radiosurgery versus observation for completely resected brain metastases: a single-centre, randomised, controlled, phase 3 trial. Lancet Oncol. (2017) 18:1040–8. doi: 10.1016/S1470-2045(17)30414-X 28687375 PMC5560102

[5] Brown PD Ballman KV Cerhan JH Anderson SK Carrero XW Whitton AC . Postoperative stereotactic radiosurgery compared with whole brain radiotherapy for resected metastatic brain disease (NCCTG N107C/CEC·3): a multicentre, randomised, controlled, phase 3 trial. Lancet Oncol. (2017) 18:1049–60. doi: 10.1016/S1470-2045(17)30441-2 28687377 PMC5568757

[6] Redmond KJ De Salles AAF Fariselli L Levivier M Ma L Paddick I . Stereotactic radiosurgery for postoperative metastatic surgical cavities: A critical review and International Stereotactic Radiosurgery Society practice guidelines. Int J Radiat Oncol Biol Phys. (2021) 111:68–80. doi: 10.1016/j.ijrobp.2021.04.016 33891979

[7] Hahnemann L Krämer A Fink C Jungk C Thomas M Christopoulos P . Fractionated stereotactic radiotherapy of intracranial postoperative cavities after resection of brain metastases - Clinical outcome and prognostic factors. Clin Transl Radiat Oncol. (2024) 46:100782. doi: 10.1016/j.ctro.2024.100782 38694237 PMC11061678

[8] Prabhu RS Dhakal R Vaslow ZK Dan T Mishra MV Murphy ES . Preoperative radiosurgery for resected brain metastases: The PROPS-BM multicenter cohort study. Int J Radiat Oncol Biol Phys. (2021) 111:764–72. doi: 10.1016/j.ijrobp.2021.05.124 34058254

[9] Maroufi SF Fallahi MS Maroufi SP Kassaeyan V Palmisciano P Sheehan JP . Preoperative versus postoperative stereotactic radiosurgery for brain metastases: a systematic review and meta-analysis of comparative studies. Neurosurg Rev. (2025) 48:16. doi: 10.1007/s10143-024-03166-6 39743641

[10] Dharnipragada R Dusenbery K Ferreira C Sharma M Chen CC . Preoperative versus postoperative radiosurgery of brain metastases: a meta-analysis. World Neurosurg. (2024) 182:35–41. doi: 10.1016/j.wneu.2023.10.131 37918565

[11] Lacny S Wilson T Clement F Roberts DJ Faris P Ghali WA . Kaplan-Meier survival analysis overestimates cumulative incidence of health-related events in competing risk settings: a meta-analysis. J Clin Epidemiol. (2018) 93:25–35. doi: 10.1016/j.jclinepi.2017.10.006 29045808

[12] Page MJ McKenzie JE Bossuyt PM Boutron I Hoffmann TC Mulrow CD . The PRISMA 2020 statement: an updated guideline for reporting systematic reviews. BMJ. (2021) 372:n71. doi: 10.1136/bmj.n71 33782057 PMC8005924

[13] Hutton B Salanti G Caldwell DM Chaimani A Schmid CH Cameron C . The PRISMA extension statement for reporting of systematic reviews incorporating network meta-analyses of health care interventions: checklist and explanations. Ann Intern Med. (2015) 162:777–84. doi: 10.7326/M14-2385 26030634

[14] Sterne JAC Hernán MA Reeves BC Savović J Berkman ND Viswanathan M . ROBINS-I: a tool for assessing risk of bias in non-randomised studies of interventions. BMJ. (2016) 355:i4919. doi: 10.1136/bmj.i4919 27733354 PMC5062054

[15] Chaimani A Caldwell DM Li T Higgins JPT Salanti G . Chapter 11: Undertaking network meta-analyses. In: Higgins JPT Thomas J Chandler J , editors. Cochrane Handbook for Systematic Reviews of Interventions. Version 6.5. Chichester: Cochrane (2024).

[16] Page MJ Higgins JPT Sterne JAC . Chapter 13: Assessing risk of bias due to missing evidence in a meta-analysis. In: Higgins JPT Thomas J Chandler J , editors. Cochrane Handbook for Systematic Reviews of Interventions. Version 6.5. Chichester: Cochrane (2024).

[17] Puhan MA Schünemann HJ Murad MH Li T Brignardello-Petersen R Singh JA . A GRADE Working Group approach for rating the quality of treatment effect estimates from network meta-analysis. BMJ. (2014) 349:g5630. doi: 10.1136/bmj.g5630 25252733

[18] Nikolakopoulou A Higgins JPT Papakonstantinou T Chaimani A Del Giovane C Egger M . CINeMA: An approach for assessing confidence in the results of a network meta-analysis. PloS Med. (2020) 17:e1003082. doi: 10.1371/journal.pmed.1003082 32243458 PMC7122720

[19] Mallela AN Iheagwara UK Fogg D Anthony A Gersey ZC Zhang X . Preoperative stereotactic radiosurgery for cerebral metastases: safe, effective, and decreases steroid dependency. J Neurosurg. (2024) 141:1332–42. doi: 10.3171/2024.2.JNS232426 38728761

[20] Eaton BR LaRiviere MJ Kim S Prabhu RS Patel K Kandula S . Hypofractionated radiosurgery has a better safety profile than single fraction radiosurgery for large resected brain metastases. J Neuro-Oncol. (2015) 123:103–11. doi: 10.1007/s11060-015-1767-4 25862006

[21] El Shafie RA Bernhardt D Welzel T Schiele A Schmitt D Thalmann P . Hypofractionated stereotactic radiotherapy versus whole-brain radiotherapy following brain metastasis resection: results of the ESTRON randomized phase 2 trial. Neuro Oncol. (2025) 27:3224–36. doi: 10.1093/neuonc/noaf182 40747537 PMC12916734

[22] Kutuk T Zhang Y Akdemir EY Yarlagadda S Tolakanahalli R Hall MD . Comparative evaluation of outcomes amongst different radiosurgery management paradigms for patients with large brain metastasis. J Neuro-Oncol. (2024) 169:105–17. doi: 10.1007/s11060-024-04706-2 38837019

[23] Patel KR Burri SH Asher AL Crocker IR Fraser RW Zhang C . Comparing preoperative with postoperative stereotactic radiosurgery for resectable brain metastases: a multi-institutional analysis. Neurosurgery. (2016) 79:279–85. doi: 10.1227/NEU.0000000000001096 26528673

[24] Minniti G Tini P Navarria P Raza G Della Pepa GM Chiesa S . Clinical outcomes of three-fraction versus five-fraction stereotactic radiosurgery for resected brain metastases. J Neuro-Oncol. (2026) 176:175. doi: 10.1007/s11060-025-05357-7 41563519 PMC12823750

[25] Perlow HK Hennings S Bradshaw J Reddy S Luu S Matsui JK . Preoperative versus postoperative fractionated stereotactic radiation therapy: a single-institution analysis of 534 resected metastases. Int J Radiat Oncol Biol Phys. (2026) 125:502–10. doi: 10.1016/j.ijrobp.2025.08.010 40825402

[26] Yeboa DN Li J Lin R Prabhu SS Beckham TH Woodhouse K . Therapy, safety, and logistics of preoperative vs postoperative stereotactic radiation therapy: a preliminary analysis of a randomized clinical trial. JAMA Oncol. (2025) 11:890–9. doi: 10.1001/jamaoncol.2025.1770 40531511 PMC12177721

[27] Kretzschmar L Gabrys H Joye A Kraft J Guckenberger M Andratschke N . Postoperative stereotactic radiosurgery (SRS) vs hypofractionated stereotactic radiotherapy (SRT) for resected brain metastases—a single centre analysis. Clin Exp Metastasis. (2025) 42:16. doi: 10.1007/s10585-025-10334-5 39928239 PMC11811445

[28] Kocher M Soffietti R Abacioglu U Villà S Fauchon F Baumert BG . Adjuvant whole-brain radiotherapy versus observation after radiosurgery or surgical resection of one to three cerebral metastases: results of the EORTC 22952-26001 study. J Clin Oncol. (2011) 29:134–41. doi: 10.1200/JCO.2010.30.1655 21041710 PMC3058272

[29] Hong AM Fogarty GB Dolven-Jacobsen K Burmeister BH Lo SN Haydu LE . Adjuvant whole-brain radiation therapy compared with observation after local treatment of melanoma brain metastases: a multicenter, randomized phase III trial. J Clin Oncol. (2019) 37:3132–41. doi: 10.1200/JCO.19.01414 31553661

[30] Patel KR Prabhu RS Kandula S Oliver DE Kim S Hadjipanayis C . Intracranial control and radiographic changes with adjuvant radiation therapy for resected brain metastases: whole brain radiotherapy versus stereotactic radiosurgery alone. J Neuro-Oncol. (2014) 120:657–63. doi: 10.1007/s11060-014-1601-4 25189789

[31] Patel KR Burri SH Boselli D Symanowski JT Asher AL Sumrall A . Comparing pre-operative stereotactic radiosurgery (SRS) to post-operative whole brain radiation therapy (WBRT) for resectable brain metastases: a multi-institutional analysis. J Neuro-Oncol. (2017) 131:611–8. doi: 10.1007/s11060-016-2334-3 28000105

[32] Broemme J Abu-Isa J Kottke R Beck J Wiest R Malthaner M . Adjuvant therapy after resection of brain metastases: frameless image-guided LINAC-based radiosurgery and stereotactic hypofractionated radiotherapy. Strahlenther Onkol. (2013) 189:765–70. doi: 10.1007/s00066-013-0409-z 23934329

[33] Hajikarimloo B Habibi MA Mohammadzadeh I Tos SM Mortezaei A Behzadi S . Neoadjuvant stereotactic radiosurgery for brain metastases: a systematic review and meta-analysis. Neuro-Oncol Pract. (2026) 13:48–59. doi: 10.1093/nop/npaf086 41798132 PMC12965656

[34] Sterne JAC Savović J Page MJ Elbers RG Blencowe NS Boutron I . RoB 2: A revised Cochrane risk-of-bias tool for randomized trials. Version 2. Cochrane. London: Cochrane (2019).

