# Supplementary material for: Preoperative versus postoperative single-fraction or fractionated stereotactic radiosurgery for resected brain metastases: a systematic review and network meta-analysis
Source: Front Oncol. 2026 Oct 14;16:1848924. doi: 10.3389/fonc.2026.1848924 (PMC13713366; doi:10.3389/fonc.2026.1848924)
Supplement: Supplementary file 1 [file DataSheet2.docx]

### Supplementary Figure S1. Twelve-month absolute risks of local failure, leptomeningeal disease, and radiation necrosis across included studies.

### Study-specific 12-month absolute risk estimates are shown for (A) local failure (LF), (B) leptomeningeal disease (LMD), and (C) radiation necrosis (RN), stratified by treatment strategy, analysis unit, and estimation method. Kaplan–Meier estimates and competing-risk cumulative-incidence estimates are presented as originally reported and were not pooled or treated as equivalent measures. Patient-level and lesion-level estimates were also retained separately. RN estimates include any-grade, symptomatic, or grade ≥2 RN as defined in the individual studies, and these definitions were not considered interchangeable. The figure is intended to provide a descriptive overview of study-specific absolute risks rather than pooled comparative treatment effects. CI, confidence interval; FSRT, fractionated stereotactic radiotherapy; LMD, leptomeningeal disease; RN, radiation necrosis; SRS, stereotactic radiosurgery; SRT, stereotactic radiotherapy; WBRT, whole-brain radiotherapy.

****Supplementary Figure S2. Robustness and evidence-path analyses of the exploratory local-failure network.**
(A)** Evidence-path and leave-one-study-out analysis of the primary local-failure network. The network comprised four treatment nodes connected by three direct comparisons, each informed by a single study. Removal of any contributing study disconnected the network or eliminated one treatment node, preventing re-estimation of the complete network and its indirect comparisons. **(B)** Sensitivity analyses evaluating the dependence of indirect estimates on the evidence informing the postoperative single-fraction versus fractionated-treatment edge and on additional uncertainty. Replacement of the patient/cavity-level estimate from Eaton et al. with the lesion-level estimate from Kretzschmar et al. preserved network connectivity but changed the direction of selected indirect estimates. Prespecified values of τ on the log-HR scale were used to examine the effect of additional uncertainty on confidence intervals and were not interpreted as empirical estimates of between-study heterogeneity. These analyses were intended as robustness and stress tests of the sparse tree-shaped network rather than as alternative primary models. CI, confidence interval; FSRT, fractionated stereotactic radiotherapy; HR, hazard ratio; SRS, stereotactic radiosurgery; SRT, stereotactic radiotherapy.

**Supplementary Figure S3. Risk-of-bias assessment of the included comparative studies.
(A) Risk-of-bias assessment of the six randomized controlled trials using the Cochrane Risk of Bias 2 (RoB 2) tool. Judgments are shown across the domains of randomization process, deviations from intended interventions, missing outcome data, measurement of the outcome, and selection of the reported result, together with the overall risk-of-bias judgment. All six randomized trials were judged to have some concerns overall. (B) Risk-of-bias assessment of the ten non-randomized comparative studies using the Risk Of Bias In Non-randomized Studies of Interventions (ROBINS-I) tool. Judgments are shown across the domains of confounding, participant selection, classification of interventions, deviations from intended interventions, missing data, measurement of outcomes, and selection of the reported result, together with the overall judgment. All ten non-randomized studies were judged to be at serious overall risk of bias, with residual confounding related to treatment selection representing the principal concern. Detailed study-level judgments and supporting rationale are provided in Supplementary Table S8.**

**Supplementary Table S1. Database and registry search strategies used for study identification**

| Source | Search period | Search strategy | Restrictions/filters | Records retrieved |
| --- | --- | --- | --- | --- |
| **PubMed/MEDLINE** | Historical: 1 Jan 2000–31 Dec 2024; update: 1 Jan 2025–12 Aug 2026 | See full strategy below | Title/Abstract fields; no language restriction; no study-design, outcome, or Humans filter | Historical: 2,149; Update: 272 |
| **Web of Science Core Collection** | Historical: 2000–2024; update: 2025–12 Aug 2026 | See full strategy below | Topic (TS); no language or document-type restriction; no study-design or outcome filter | Historical: 2,994; Update: 284 |
| **Cochrane CENTRAL** | Historical: publication years 2000–2024; update: publication years 2025–2026, checked through 12 Aug 2026 | See full strategy below | Title, abstract, and keywords; CENTRAL Trials only; no language or outcome restriction | Historical: 350; Update: 32 |
| **ClinicalTrials.gov** | Historical: first posted 1 Jan 2000–31 Dec 2024; update: first posted or last updated 1 Jan 2025–12 Aug 2026 | See full strategy below | No restriction by study type or recruitment status; no outcome filter | Historical: 234; Update: 114* |

**Notes:**

PubMed/MEDLINE:( ("brain metastasis"[Title/Abstract] OR "brain metastases"[Title/Abstract] OR "cerebral metastasis"[Title/Abstract] OR "cerebral metastases"[Title/Abstract] OR "intracranial metastasis"[Title/Abstract] OR "intracranial metastases"[Title/Abstract]) AND (resect*[Title/Abstract] OR surg*[Title/Abstract] OR craniotom*[Title/Abstract] OR metastasectom*[Title/Abstract] OR "resection cavity"[Title/Abstract] OR "surgical cavity"[Title/Abstract] OR preoperat*[Title/Abstract] OR postoperat*[Title/Abstract] OR perioperat*[Title/Abstract] OR neoadjuvant[Title/Abstract] ) AND ( radiosurg*[Title/Abstract] OR "stereotactic radiosurgery"[Title/Abstract] OR SRS[Title/Abstract] OR "stereotactic radiotherapy"[Title/Abstract] OR SRT[Title/Abstract] OR "fractionated stereotactic"[Title/Abstract] OR "hypofractionated stereotactic"[Title/Abstract] OR FSRT[Title/Abstract] OR fSRS[Title/Abstract] OR HFSRT[Title/Abstract] OR "whole brain radiotherapy"[Title/Abstract] OR "whole-brain radiotherapy"[Title/Abstract] OR WBRT[Title/Abstract] ))

Web of Science Core Collection:TS=( ( "brain metastas " OR "cerebral metastas*" OR "intracranial metastas*") AND ( resect* OR surg* OR craniotom OR metastasectom* OR "resection cavity" OR "surgical cavity" OR preoperat* OR postoperat* OR perioperat* OR neoadjuvant ) AND ( radiosurg* OR "stereotactic radiosurgery" OR SRS OR "stereotactic radiotherapy" OR SRT OR "fractionated stereotactic" OR "hypofractionated stereotactic" OR FSRT OR fSRS OR HFSRT OR "whole brain radiotherapy" OR "whole-brain radiotherapy" OR WBRT ) )

Cochrane Central Register of Controlled Trials (CENTRAL):#1 ("brain metastas" OR "cerebral metastas*" OR "intracranial metastas*"):ti,ab,kw

#2 (resect* OR surg* OR craniotom* OR metastasectom* OR "resection cavity" OR "surgical cavity" OR preoperat* OR postoperat* OR perioperat* OR neoadjuvant):ti,ab,kw #3 (radiosurg* OR "stereotactic radiosurgery" OR SRS OR "stereotactic radiotherapy" OR SRT OR "fractionated stereotactic" OR "hypofractionated stereotactic" OR FSRT OR fSRS OR HFSRT OR "whole brain radiotherapy" OR "whole-brain radiotherapy" OR WBRT):ti,ab,kw #4 #1 AND #2 AND #3

ClinicalTrials.gov:("brain metastasis" OR "brain metastases") AND (resection OR surgery) AND ( radiosurgery OR "stereotactic radiotherapy" OR SRS OR SRT OR FSRT OR fSRS OR HFSRT OR "whole brain radiotherapy" OR WBRT )

Historical-period filter:AREA[StudyFirstPostDate]RANGE[01/01/2000, 12/31/2024]

Update-period filter:AREA[StudyFirstPostDate]RANGE[01/01/2025, 08/12/2026] OR AREA[LastUpdatePostDate]RANGE[01/01/2025, 08/12/2026]

The ClinicalTrials.gov update search included records first posted or last updated during the specified period and could therefore overlap with records retrieved in the historical search. Accordingly, historical and update retrieval counts for ClinicalTrials.gov should not be interpreted as mutually exclusive unique records.

CENTRAL retrieval counts were verified from the Cochrane Search Manager results page and retained screenshots. Complete CENTRAL records could not be exported because of institutional-access restrictions.

Retrieval counts represent raw database- or registry-specific search results before deduplication. Records retrieved across sources and search periods were deduplicated before screening; 415 unique records subsequently underwent title and abstract screening. Therefore, the database-specific retrieval counts shown above should not be summed and interpreted as the number of unique records screened.

**Supplementary Table S2. Twelve-month absolute risks: local failure**

| **Study** | **Design** | **Outcome** | **Treatment** | **n** | **12-month risk** | **95% CI lower** | **95% CI upper** | **Estimation method** | **Analysis unit** | **Estimand** | **Caveat** | **Outcome group** |
| --- | --- | --- | --- | --- | --- | --- | --- | --- | --- | --- | --- | --- |
| Mahajan et al. 2017 | Randomized trial | Local failure | Observation | 65 | 57.0% | 41.0% | 69.0% | Kaplan–Meier | Patient | 1-KM freedom from local recurrence |  | LC |
| Mahajan et al. 2017 | Randomized trial | Local failure | Postoperative single-fraction SRS | 63 | 28.0% | 13.0% | 40.0% | Kaplan–Meier | Patient | 1-KM freedom from local recurrence |  | LC |
| El Shafie et al. 2025 (ESTRON) | Randomized trial | Local failure | Postoperative 7-fraction FSRT | 27 | 2.0% | 0.2% | 17.4% | Competing-risk/time-to-event | Patient | competing-risk cumulative incidence | Do not convert to integer events | LC |
| El Shafie et al. 2025 (ESTRON) | Randomized trial | Local failure | WBRT | 27 | 5.3% | 1.2% | 22.4% | Competing-risk/time-to-event | Patient | competing-risk cumulative incidence | Do not convert to integer events | LC |
| Mallela et al. 2024 | Retrospective cohort | Local failure | Preoperative single-fraction SRS | 26 | 22.8% | 2.7% | 38.7% | Kaplan–Meier | Patient | 1-KM local tumor control | Matched cohort; use HR primary | LC |
| Mallela et al. 2024 | Retrospective cohort | Local failure | Postoperative single-fraction SRS | 30 | 17.5% | 0.0% | 38.8% | Kaplan–Meier | Patient | 1-KM local tumor control | Matched cohort; use HR primary | LC |
| Patel et al. 2015 | Retrospective cohort | Local failure | Predominantly single-fraction preoperative SRS | 66 | 15.9% |  |  | Competing-risk/time-to-event | Patient | competing-risk cumulative incidence | Mixed comparator and cohort overlap | LC |
| Patel et al. 2015 | Retrospective cohort | Local failure | Mixed postoperative SRS | 114 | 12.6% |  |  | Competing-risk/time-to-event | Patient | competing-risk cumulative incidence | Mixed comparator and cohort overlap | LC |
| Eaton et al. 2015 | Retrospective cohort | Local failure | Postoperative 3–5-fraction FSRT/SRS | 36 | 25.6% | 12.0% | 42.0% | Competing-risk/time-to-event | Patient | competing-risk cumulative incidence | Use reported HR; strong imbalance | LC |
| Eaton et al. 2015 | Retrospective cohort | Local failure | Postoperative single-fraction SRS | 40 | 27.2% | 14.0% | 42.0% | Competing-risk/time-to-event | Patient | competing-risk cumulative incidence | Use reported HR; strong imbalance | LC |
| Minniti et al. 2026 | Retrospective cohort | Local failure | Postoperative 3-fraction SRS | 103 | 7.0% | 2.0% | 13.0% | Competing-risk/time-to-event | Lesion | competing-risk cumulative incidence | Lesion-level n; regimen chosen by clinician | LC |
| Minniti et al. 2026 | Retrospective cohort | Local failure | Postoperative 5-fraction FSRT/SRS | 99 | 9.8% | 5.5% | 12.5% | Competing-risk/time-to-event | Lesion | competing-risk cumulative incidence | Lesion-level n; published CI appears unusually narrow and requires checking against figure | LC |
| Perlow et al. 2026 | Retrospective cohort | Local failure | Preoperative 3–5-fraction FSRT | 235 | 2.4% | 0.9% | 5.2% | Competing-risk/time-to-event | Lesion | competing-risk cumulative incidence | Lesion-level analysis; repeated lesions not adjusted; strong volume/fraction/year imbalance | LC |
| Perlow et al. 2026 | Retrospective cohort | Local failure | Postoperative 3–5-fraction FSRT/SRS | 299 | 2.4% | 1.1% | 4.7% | Competing-risk/time-to-event | Lesion | competing-risk cumulative incidence | Lesion-level analysis; repeated lesions not adjusted; strong volume/fraction/year imbalance | LC |
| Kutuk et al. 2024 | Retrospective cohort | Local failure | Preoperative single-fraction SRS | 47 | 7.0% | 2.0% | 17.0% | Competing-risk/time-to-event | Lesion | competing-risk cumulative incidence | Large lesions >=2 cm; unadjusted four-group comparison | LC |
| Kutuk et al. 2024 | Retrospective cohort | Local failure | Postoperative 5-fraction FSRT/SRS | 74 | 8.0% | 3.0% | 16.0% | Competing-risk/time-to-event | Lesion | competing-risk cumulative incidence | Large lesions >=2 cm; postoperative regimen 30 Gy/5 fractions | LC |

Notes: CI, confidence interval; FSRT, fractionated stereotactic radiotherapy; LMD, leptomeningeal disease; RN, radiation necrosis; SRS, stereotactic radiosurgery; WBRT, whole-brain radiotherapy. Blank confidence limits indicate that arm-level limits were not reported or were not retained in the source extraction. Patient-level and lesion-level estimates must not be combined.

**Supplementary Table S3. Within-study twelve-month absolute risk differences: local failure**

| **Study** | **Design** | **Outcome** | **Treatment 1** | **Treatment 2** | **Risk 1** | **Risk 2** | **Absolute risk difference** | **Estimation method** | **Analysis unit** | **Interpretation rule** | **Outcome group** |
| --- | --- | --- | --- | --- | --- | --- | --- | --- | --- | --- | --- |
| Eaton et al. 2015 | Retrospective cohort | Local failure | Postoperative 3–5-fraction FSRT/SRS | Postoperative single-fraction SRS | 25.6% | 27.2% | -1.6% | Competing-risk/time-to-event | Patient | Positive = higher risk with Treatment 1 | LC |
| El Shafie et al. 2025 (ESTRON) | Randomized trial | Local failure | Postoperative 7-fraction FSRT | WBRT | 2.0% | 5.3% | -3.3% | Competing-risk/time-to-event | Patient | Positive = higher risk with Treatment 1 | LC |
| Kutuk et al. 2024 | Retrospective cohort | Local failure | Preoperative single-fraction SRS | Postoperative 5-fraction FSRT/SRS | 7.0% | 8.0% | -1.0% | Competing-risk/time-to-event | Lesion | Positive = higher risk with Treatment 1 | LC |
| Mahajan et al. 2017 | Randomized trial | Local failure | Observation | Postoperative single-fraction SRS | 57.0% | 28.0% | 29.0% | Kaplan–Meier | Patient | Positive = higher risk with Treatment 1 | LC |
| Mallela et al. 2024 | Retrospective cohort | Local failure | Preoperative single-fraction SRS | Postoperative single-fraction SRS | 22.8% | 17.5% | 5.3% | Kaplan–Meier | Patient | Positive = higher risk with Treatment 1 | LC |
| Minniti et al. 2026 | Retrospective cohort | Local failure | Postoperative 3-fraction SRS | Postoperative 5-fraction FSRT/SRS | 7.0% | 9.8% | -2.8% | Competing-risk/time-to-event | Lesion | Positive = higher risk with Treatment 1 | LC |
| Patel et al. 2015 | Retrospective cohort | Local failure | Predominantly single-fraction preoperative SRS | Mixed postoperative SRS | 15.9% | 12.6% | 3.3% | Competing-risk/time-to-event | Patient | Positive = higher risk with Treatment 1 | LC |
| Perlow et al. 2026 | Retrospective cohort | Local failure | Preoperative 3–5-fraction FSRT | Postoperative 3–5-fraction FSRT/SRS | 2.4% | 2.4% | 0.0% | Competing-risk/time-to-event | Lesion | Positive = higher risk with Treatment 1 | LC |

Notes: These are study-specific descriptive contrasts and were not pooled. Their signs depend on the displayed treatment order and do not imply statistical significance or causal superiority. Abbreviations: FSRT, fractionated stereotactic radiotherapy; LMD, leptomeningeal disease; RN, radiation necrosis; SRS, stereotactic radiosurgery; WBRT, whole-brain radiotherapy.

**Supplementary Table S4. Study-design-stratified relative effects: local failure**

| **Study** | **Study design** | **Treatment 1** | **Treatment 2** | **Effect measure** | **Adjustment** | **Estimate** | **95% CI lower** | **95% CI upper** | **Analysis unit** | **Unit group** | **Evidence role** | **Study note** | **Direction** |
| --- | --- | --- | --- | --- | --- | --- | --- | --- | --- | --- | --- | --- | --- |
| Mallela et al. 2024 | Propensity-score-matched study | Preoperative single-fraction SRS | Postoperative single-fraction SRS | HR | Adjusted | 1.57 | 0.38 | 6.49 | Patient | patient/cavity-level | Primary evidence set | adjusted HR | A vs B |
| Kretzschmar et al. 2025 | Retrospective cohort | Postoperative fractionated SRT/SRS | Postoperative single-fraction SRS | HR | Adjusted | 3.38 | 0.43 | 26.60 | Lesion | lesion-level | Sensitivity analysis | adjusted HR; PTV adjusted | C vs B |
| Perlow et al. 2026 | Retrospective cohort | Postoperative fractionated SRT/SRS | Preoperative 3–5-fraction FSRT | SHR | Adjusted | 0.84 | 0.30 | 2.33 | Lesion | lesion-level | Extension analysis | adjusted SHR | C vs A2 |
| Mahajan et al. 2017 | Randomized trial | Postoperative single-fraction SRS | Observation | HR | Reported estimate | 0.46 | 0.24 | 0.88 | Patient | patient/cavity-level | Primary evidence set | reported HR | B vs E |
| Eaton et al. 2015 | Retrospective cohort | Postoperative single-fraction SRS | Postoperative fractionated SRT/SRS | HR | Reported estimate | 0.82 | 0.09 | 7.32 | Patient/cavity | patient/cavity-level | Primary evidence set | reported HR | B vs C |

Notes:CI, confidence interval; FSRT, fractionated stereotactic radiotherapy; HR, hazard ratio; LMD, leptomeningeal disease; PTV, planning target volume; RN, radiation necrosis; SHR, subdistribution hazard ratio; SRS, stereotactic radiosurgery. Estimates are reproduced in their originally extracted treatment direction. Evidence roles indicate their use in the reconstructed analyses and do not represent certainty ratings.

**Supplementary Table S5. Direct and indirect evidence pathways**

| **Comparison** | **Evidence type** | **Path edge** | **Contributing study** | **Uncertainty share** | **Interpretive note** |
| --- | --- | --- | --- | --- | --- |
| A vs B | direct | A–B | Mallela et al. 2024 | 100.0% | Variance share along the unique tree path; not a formal net contribution matrix |
| B vs C | direct | B–C | Eaton et al. 2015 | 100.0% | Variance share along the unique tree path; not a formal net contribution matrix |
| B vs E | direct | B–E | Mahajan et al. 2017 | 100.0% | Variance share along the unique tree path; not a formal net contribution matrix |
| A vs C | indirect only | A–B | Mallela et al. 2024 | 29.6% | Variance share along the unique tree path; not a formal net contribution matrix |
| A vs C | indirect only | B–C | Eaton et al. 2015 | 70.4% | Variance share along the unique tree path; not a formal net contribution matrix |
| A vs E | indirect only | A–B | Mallela et al. 2024 | 82.7% | Variance share along the unique tree path; not a formal net contribution matrix |
| A vs E | indirect only | B–E | Mahajan et al. 2017 | 17.3% | Variance share along the unique tree path; not a formal net contribution matrix |
| C vs E | indirect only | B–C | Eaton et al. 2015 | 91.9% | Variance share along the unique tree path; not a formal net contribution matrix |
| C vs E | indirect only | B–E | Mahajan et al. 2017 | 8.1% | Variance share along the unique tree path; not a formal net contribution matrix |

Notes:A, preoperative single-fraction SRS; B, postoperative single-fraction SRS; C, postoperative three- to five-fraction FSRT/SRS; E, observation. Uncertainty shares describe the unique tree path and are not a formal net contribution matrix.

**Supplementary Table S6. Clinical heterogeneity and transitivity matrix**

| **Study** | **Design** | **Comparison** | **Analysis unit** | **Size/selection** | **Dose/fractionation** | **Follow-up** | **Immunotherapy** | **Primary tumours** | **Outcome method** | **Transitivity concern** |
| --- | --- | --- | --- | --- | --- | --- | --- | --- | --- | --- |
| Mahajan et al. 2017 | RCT | B vs E | patient | resection cavity ≤4 cm | post-op single-fraction SRS vs observation | median 11.1 months | NR | stratified by histology | time to local recurrence; KM | low-moderate |
| Mallela et al. 2024 | prospective propensity-matched | A vs B | patient | no volumetric cutoff; unequal GTV remained | single-fraction pre/post SRS | NR in matrix extraction | prior immunotherapy 12.5%; arm imbalance possible | mixed; matched on oncological history | LTC time-to-event HR | serious residual volume confounding |
| Eaton et al. 2015 | retrospective | B vs C | cavity/patient | large cavities; baseline imbalance | post-op single vs 3–5 fractions | NR in matrix extraction | NR | mixed | competing-risk cumulative incidence/HR | serious volume/RPA imbalance |
| Kretzschmar et al. 2025 | retrospective | C(mostly 6F) vs B | lesion/cavity | fractionation strongly selected by cavity volume | post-op single vs mostly 6 fractions | NR in matrix extraction | NR | mixed | adjusted HR; lesion/cavity analysis | serious confounding by indication |
| Brown et al. 2017 | RCT | B vs D | patient | resected cavity | post-op SRS vs WBRT | NR in matrix extraction | NR | mixed | competing-risk cumulative incidence | different comparator modality |
| El Shafie et al. 2025 | phase-2 RCT | C(7F) vs D | patient | resected patients | 7-fraction HFSRT vs WBRT | NR in matrix extraction | concurrent immunotherapy imbalanced | mixed | competing-risk cumulative incidence | 7F node and immunotherapy imbalance |
| Kutuk et al. 2024 | retrospective | A vs C(5F) | lesion | large lesions ≥2 cm | pre-op single vs post-op 30 Gy/5F | median 14 months | reported and heterogeneous | lung most common | Gray test; death competing risk | large-lesion and lesion-level restriction |
| Patel et al. 2015 | retrospective multicentre | A vs B_mixed | patient | mixed postoperative cohort | pre-op mostly single vs post-op mixed | NR in matrix extraction | NR | mixed | competing-risk cumulative incidence/SHR | mixed node and overlapping cohort |
| Perlow et al. 2026 | retrospective multicentre | C vs A2 | lesion | treatment volume differed; larger lesions more often 5F | 27 Gy/3F or 30 Gy/5F pre/post | median 12 months | recorded; era/fraction imbalance | lung plurality (41%) | Gray test; death competing risk | A2 extension; lesion-level and era imbalance |
| Minniti et al. 2026 | retrospective multicentre | C3F vs C5F | patient for safety; lesion for LC | regimen selected clinically | 3 fractions vs 5 fractions | NR in matrix extraction | NR | mixed | competing-risk cumulative incidence | within-node regimen comparison |

Notes:NR, not reported in the matrix extraction. Differences across comparisons limited empirical support for transitivity; this table is a structured clinical assessment rather than a statistical test.

**Supplementary Table S7. Study-level synthesis of secondary outcomes**

| **Study** | **Design** | **Comparison** | **Outcome** | **Time point / estimand** | **Group-specific results** | **Relative effect / P value** | **Analysis unit** | **Interpretation boundary** |
| --- | --- | --- | --- | --- | --- | --- | --- | --- |
| Kocher et al. 2011 | Phase III RCT | WBRT vs observation | Overall survival | Median OS | 10.9 vs 10.7 months | HR 0.98 (95% CI 0.78–1.24); P=0.89 | Patient | Trial included surgical and radiosurgery strata; the surgical subgroup was relevant to this review |
| Kocher et al. 2011 | Phase III RCT | WBRT vs observation | Intracranial relapse at new sites | 2-year relapse rate in surgical subgroup | 23% vs 42% | P=0.008 | Patient | Subgroup result; not pooled with differently defined distant-intracranial outcomes |
| Patel et al. 2014 | Retrospective cohort | Postoperative SRS vs WBRT | Distant brain control | Adjusted 1-year DBC | 48% vs 70% | SRS vs WBRT HR for DBF 2.14 (95% CI 1.14–4.04); P=0.02 | Patient | Non-randomized comparison; treatment selection and number of metastases may confound the estimate |
| Patel et al. 2015 | Multicentre retrospective cohort | Preoperative SRS vs postoperative SRS | Overall survival | Time-to-event analysis | NR in summary extraction | P=0.10 | Patient | Overlapping cohorts were assessed; no pooled estimate |
| Patel et al. 2015 | Multicentre retrospective cohort | Preoperative SRS vs postoperative SRS | Distant brain recurrence | Competing-risk analysis | NR in summary extraction | P=0.75 | Patient | Mixed postoperative regimens and cohort-overlap concern |
| Mahajan et al. 2017 | Phase III RCT | Postoperative SRS vs observation | Overall survival | Median OS | 17 vs 18 months | HR 1.29 (95% CI 0.84–1.98); P=0.24 | Patient | Single direct randomized comparison |
| Mahajan et al. 2017 | Phase III RCT | Postoperative SRS vs observation | Distant brain recurrence | 12-month estimate | 42% vs 33% | HR 0.81 (95% CI 0.51–1.27); P=0.35 | Patient | Outcome wording and HR direction retained as reported; not pooled |
| Brown et al. 2017 | Phase III RCT | Postoperative SRS vs WBRT | Overall survival | Median OS | 12.2 vs 11.6 months | HR 1.07 (95% CI 0.76–1.50); P=0.70 | Patient | Single direct randomized comparison |
| Brown et al. 2017 | Phase III RCT | Postoperative SRS vs WBRT | Intracranial tumour progression | Median time to progression | 6.4 vs 27.5 months | HR 2.45 (95% CI 1.62–3.72); P<0.0001 | Patient | Composite intracranial progression; not equivalent to isolated distant brain failure |
| Brown et al. 2017 | Phase III RCT | Postoperative SRS vs WBRT | Wound events | Adverse-event reporting | Wound infection: 1 vs 0; wound complication: 1 vs 0 | No comparative effect reported | Patient | Very few events; no pooled estimate or equivalence claim |
| Hong et al. 2019 | Phase III RCT | WBRT vs observation | Distant intracranial failure | 12-month incidence | 42.0% vs 50.5% | OR 0.71 (95% CI 0.41–1.23); P=0.22 | Patient | Mixed local-treatment population; 64.0% vs 59.8% underwent surgery |
| Hong et al. 2019 | Phase III RCT | WBRT vs observation | Overall survival | Median OS; 12-month OS | 16.5 vs 13.0 months; 58.4% vs 54.0% | P=0.86 for median OS; P=0.89 at 12 months | Patient | Mixed local-treatment population; resected subgroup not separately estimated |
| Mallela et al. 2024 | Prospective propensity-matched cohort | Preoperative vs postoperative single-fraction SRS | Wound-healing issues | Within 3 months | 1/26 (4.5%) vs 1/30 (4.0%) | P>0.99 | Patient | Events required operative wound revision; small matched cohorts and very few events |
| El Shafie et al. 2025 (ESTRON) | Phase II RCT | Postoperative 7-fraction HFSRT vs WBRT | Intracranial progression-free survival | 12-month ic-PFS; median ic-PFS | 44.4% vs 59.3%; 4.7 vs 15.0 months | HR 1.72; P=0.080 | Patient | Seven-fraction HFSRT is a separate treatment category |
| El Shafie et al. 2025 (ESTRON) | Phase II RCT | Postoperative 7-fraction HFSRT vs WBRT | Overall survival | 1-year OS; median OS | 63.0% vs 77.8%; 17.8 vs 27.0 months | HR 1.09; P=0.336 | Patient | Small phase II trial; separate treatment component |
| El Shafie et al. 2025 (ESTRON) | Phase II RCT | Postoperative 7-fraction HFSRT vs WBRT | Treatment-related adverse events | All reported events; grade 3 events | 54 vs 115 events; 11% vs 19% grade 3 events | No pooled comparative effect | Patient | Event counts may include multiple events per patient; not a wound-specific outcome |
| Yeboa et al. 2025 | Phase III RCT | Preoperative vs postoperative SRT | Thirty-day postoperative events | Events within 30 days | 22 vs 17 events | P=0.87 | Patient | Initial safety analysis; event counts are not necessarily numbers of affected patients; oncological outcomes immature |
| Yeboa et al. 2025 | Phase III RCT | Preoperative vs postoperative SRT | Treatment logistics | Time from randomization to completion of both RT and surgery | Median 10 days (range 4–31) vs 32.5 days (19–55) | P<0.001 | Patient | Feasibility/logistics outcome rather than efficacy or oncological safety |

Notes:CI, confidence interval; DBC, distant brain control; DBF, distant brain failure; HFSRT, hypofractionated stereotactic radiotherapy; HR, hazard ratio; ic-PFS, intracranial progression-free survival; NR, not reported in the verified summary extraction; OR, odds ratio; OS, overall survival; RCT, randomized controlled trial; SRS, stereotactic radiosurgery; SRT, stereotactic radiotherapy; WBRT, whole-brain radiotherapy.

**Supplementary Table S8. Outcome-level risk-of-bias judgments**

| **Study** | **Tool** | **Outcome assessed** | **Domain 1** | **Domain 2** | **Domain 3** | **Domain 4** | **Domain 5** | **Domain 6** | **Domain 7** | **Overall judgment** | **Key rationale** |
| --- | --- | --- | --- | --- | --- | --- | --- | --- | --- | --- | --- |
| Kocher et al. 2011 | RoB 2 | Intracranial outcomes and overall survival | Low | Some concerns | Some concerns | Low | Low | NA | NA | Some concerns | Randomized trial; open-label care and incomplete outcome availability raise concerns, although objective survival outcomes are less vulnerable to measurement bias. |
| Mahajan et al. 2017 | RoB 2 | Local failure, LMD and overall survival | Low | Low | Some concerns | Some concerns | Low | NA | NA | Some concerns | Randomization was described; modified intention-to-treat exclusions and unmasked radiographic assessment introduce some concern. |
| Brown et al. 2017 | RoB 2 | Local/intracranial control, survival and adverse events | Low | Low | Some concerns | Some concerns | Low | NA | NA | Some concerns | Multicentre randomized trial with prespecified analyses; attrition and unmasked outcome assessment remain relevant for selected outcomes. |
| Hong et al. 2019 | RoB 2 | Distant intracranial failure and overall survival | Low | Low | Some concerns | Some concerns | Low | NA | NA | Some concerns | Randomized trial; incomplete intracranial follow-up and unmasked imaging assessment raise some concern. Applicability of the mixed local-treatment population was assessed separately as indirectness. |
| El Shafie et al. 2025 (ESTRON) | RoB 2 | LC, LMD, intracranial PFS, survival and toxicity | Some concerns | Low | Some concerns | Some concerns | Low | NA | NA | Some concerns | Small single-centre phase II randomized trial; limited information on allocation concealment, small numbers and unmasked outcome assessment. |
| Yeboa et al. 2025 | RoB 2 | Perioperative safety and feasibility | Low | Low | Some concerns | Some concerns | Some concerns | NA | NA | Some concerns | Randomized trial reporting an initial safety analysis; event ascertainment was unmasked and mature oncological outcomes were not yet reported. |
| Brömme et al. 2013 | ROBINS-I | LC and RN | Serious | Moderate | Moderate | Serious | Moderate | Moderate | Moderate | Serious | Retrospective treatment selection, small sample, differing fractionation by cavity characteristics and limited adjustment for confounding. |
| Patel et al. 2014 | ROBINS-I | LC and distant brain control | Serious | Moderate | Moderate | Moderate | Moderate | Moderate | Moderate | Serious | Non-randomized treatment allocation with residual prognostic imbalance despite multivariable adjustment. |
| Eaton et al. 2015 | ROBINS-I | Local failure and RN | Serious | Moderate | Moderate | Moderate | Moderate | Moderate | Moderate | Serious | Fractionation was associated with cavity size and planning factors; substantial baseline imbalance and limited confounder control. |
| Patel et al. 2015 | ROBINS-I | LC, LMD and RN | Serious | Moderate | Moderate | Moderate | Moderate | Moderate | Serious | Serious | Retrospective timing comparison, mixed postoperative regimens and potential overlap with related institutional cohorts. |
| Patel et al. 2016 | ROBINS-I | Intracranial outcomes and survival | Serious | Moderate | Moderate | Moderate | Moderate | Moderate | Serious | Serious | Multi-institutional retrospective comparison with treatment-selection bias and overlapping-cohort concern. |
| Mallela et al. 2024 | ROBINS-I | LC, LMD and adverse radiation effect | Serious | Moderate | Moderate | Moderate | Moderate | Moderate | Moderate | Serious | Propensity matching reduced measured imbalance, but residual cavity-volume and treatment-selection confounding remained. |
| Kutuk et al. 2024 | ROBINS-I | LF, LMD and RN in large lesions | Serious | Moderate | Moderate | Moderate | Moderate | Serious | Moderate | Serious | Retrospective four-strategy comparison restricted to large lesions; unadjusted operative contrast and lesion-level analysis. |
| Kretzschmar et al. 2025 | ROBINS-I | LC and RN | Serious | Moderate | Moderate | Moderate | Moderate | Serious | Moderate | Serious | Confounding by indication was substantial because fractionation depended strongly on cavity volume; lesion/cavity analysis and incomplete clustering control. |
| Minniti et al. 2026 | ROBINS-I | LC, symptomatic RN and LMD | Serious | Moderate | Moderate | Moderate | Moderate | Serious | Moderate | Serious | Clinician-selected three- versus five-fraction regimens and mixed patient/lesion analysis with residual confounding. |
| Perlow et al. 2026 | ROBINS-I | LF, nodular meningeal disease and grade ≥2 RN | Serious | Moderate | Moderate | Moderate | Serious | Serious | Moderate | Serious | Treatment era, target volume and fractionation differed between groups; lesion-level analyses did not fully account for multiple lesions per patient. |

Notes:RoB 2 domains: D1 randomization; D2 deviations; D3 missing outcome data; D4 outcome measurement; D5 selection of reported result. ROBINS-I domains: D1 confounding; D2 participant selection; D3 intervention classification; D4 deviations; D5 missing data; D6 outcome measurement; D7 selection of reported result. Judgments reflect the information available in the reports and supplements.

**Supplementary Table S9. Certainty of evidence for local-failure comparisons**

| **comparison** | **evidence** | **within_study_bias** | **reporting_bias** | **indirectness** | **imprecision** | **heterogeneity** | **incoherence** | **certainty** | **rationale** | **framework_note** |
| --- | --- | --- | --- | --- | --- | --- | --- | --- | --- | --- |
| A vs B | direct | Serious | Undetected but cannot be excluded | Serious | Very serious | Not estimable | Not applicable—no loop | Very low | Single matched nonrandomized study; wide CI and residual volume confounding | Author-side adapted GRADE/CINeMA-style judgment; not a formal CINeMA software output |
| B vs C | direct | Serious | Undetected but cannot be excluded | Serious | Very serious | Not estimable | Not applicable—no loop | Very low | Single retrospective study; very wide CI and analysis-unit concern | Author-side adapted GRADE/CINeMA-style judgment; not a formal CINeMA software output |
| B vs E | direct | Not serious | Undetected but cannot be excluded | Not serious | Serious | Not estimable | Not applicable—no loop | Moderate | Single RCT with CI excluding 1, but one-study evidence and limited precision | Author-side adapted GRADE/CINeMA-style judgment; not a formal CINeMA software output |
| A vs C | indirect | Serious | Undetected but cannot be excluded | Very serious | Very serious | Not estimable | Not testable—no loop | Very low | Entirely indirect two-edge path through heterogeneous observational studies | Author-side adapted GRADE/CINeMA-style judgment; not a formal CINeMA software output |
| A vs E | indirect | Serious | Undetected but cannot be excluded | Serious | Very serious | Not estimable | Not testable—no loop | Very low | Indirect path combines matched cohort with RCT; wide CI | Author-side adapted GRADE/CINeMA-style judgment; not a formal CINeMA software output |
| C vs E | indirect | Serious | Undetected but cannot be excluded | Serious | Very serious | Not estimable | Not testable—no loop | Very low | Indirect path combines retrospective cohort with RCT; very wide CI | Author-side adapted GRADE/CINeMA-style judgment; not a formal CINeMA software output |

Notes:A, preoperative single-fraction SRS; B, postoperative single-fraction SRS; C, postoperative three- to five-fraction FSRT/SRS; E, observation. Heterogeneity was not estimable and incoherence was not testable because the network was a three-edge tree without a closed loop.
